# Oncogenic epidermal growth factor receptor signal-induced histone deacetylation suppresses chemokine gene expression in human lung adenocarcinoma

**DOI:** 10.1038/s41598-023-32177-4

**Published:** 2023-03-29

**Authors:** Hidetoshi Sumimoto, Atsushi Takano, Tomoyuki Igarashi, Jun Hanaoka, Koji Teramoto, Yataro Daigo

**Affiliations:** 1 Department of Medical Oncology and Cancer Center, Shiga University of Medical Science, Otsu, Shiga Japan; 2 Center for Advanced Medicine Against Cancer, Shiga University of Medical Science, Otsu, Shiga Japan; 3 Center for Antibody and Vaccine Therapy, Institute of Medical Science, Research Hospital, The University of Tokyo, Tokyo, Japan; 4 Department of Surgery, Shiga University of Medical Science, Otsu, Shiga Japan

**Keywords:** Cancer, Immunology, Molecular biology, Oncology

## Abstract

*Epidermal growth factor receptor* (*EGFR*)-mutated (mt) lung adenocarcinoma (LA) is refractory to immune checkpoint inhibitors (ICIs). However, the mechanisms have not been fully elucidated. CD8^+^ T cell infiltration was significantly lower in *EGFR*-mt than in *EGFR*-wild-type LA, which was associated with suppression of chemokine expression. Since this T cell-deserted tumor microenvironment may lead to the refractoriness of ICIs against *EGFR*-mt LA, we investigated the mechanism by focusing on the regulation of chemokine expression. The expression of C-X-C motif ligand (CXCL) 9, 10 and 11, which constitute a gene cluster on chromosome 4, was suppressed under EGFR signaling. The assay for transposase-accessible chromatin with high-throughput sequencing (ATAC-seq) revealed open chromatin peaks near this gene cluster following EGFR-tyrosine kinase inhibitor (TKI) treatment. The histone deacetylase (HDAC) inhibitor recovered the expression of *CXCL9*, *10* and *11* in *EGFR*-mt LA. Nuclear HDAC activity, as well as histone H3 deacetylation, were dependent on oncogenic EGFR signaling. Furthermore, the Cleavage Under Targets and Tagmentation (CUT & Tag) assay revealed a histone H3K27 acetylation peak at 15 kb upstream of *CXCL11* after treatment with EGFR-TKI, which corresponded to one of the open chromatin peaks detected by ATAC-seq. The data suggest that EGFR-HDAC axis mediates silencing of the chemokine gene cluster through chromatin conformational change, which might be relevant to the ICI resistance by creating T cell-deserted tumor microenvironment. Targeting this axis may develop a new therapeutic strategy to overcome the ICI resistance of *EGFR*-mt LA.

## Introduction

Since the early 2010s, the success of clinical trials using immune checkpoint inhibitors (ICIs), such as anti-programmed death-1 (PD-1) monoclonal antibodies (mAbs), has changed the clinical practices in cancer treatment^1–3^. Additionally, new clinical pieces of evidence, which reveal the efficacy of ICIs against various tumors and in different settings, are emerging^4^. However, some patients with cancer are resistant to ICIs. Various strategies have been devised to mitigate the resistance of cancers to ICIs.

Early clinical studies have revealed that human lung adenocarcinoma (LA) harboring oncogenic *EGFR* mutations is resistant to ICIs^5–7^. Hence, patients harboring *EGFR* mutations have been likely to be excluded from most of the ongoing ICI clinical trials on human LA. Oncogenic *EGFR* mutations were observed in approximately 50% of LA cases in a Japanese population^8^. The elucidation of the resistance mechanisms of *EGFR*-mutated (*EGFR*-mt) LA can contribute to the development of therapeutic strategies to overcome ICI resistance in *EGFR*-mt LA and facilitate an improved understanding of ICI mechanisms in cancer immunity. Several previous reports have suggested the potential refractory mechanisms of *EGFR*-mt LA for ICIs^9–11^, however, no clinical trials to mitigate the resistance have been performed. Since we noticed the significant decrease of *CD8A* mRNA in *EGFR*-mt LA from TCGA data, we further attempted to clarify the underlying mechanism.

C-X-C motif ligand (CXCL) chemokines, CXCL9, 10, and 11, are IFN-responsive and associated with the recruitment of activated Th1 cells, and have pleiotropic functions against T cells, such as migration, differentiation and activation, and play crucial roles in immune activation and tumor rejection in TME via its cognate receptor, CXCR3^12^, and anti-tumor effect of anti-PD-1 therapy decreases in *CXCR3* knock-out mice^13^.

We have confirmed that the number of CD8^+^ T cells in *EGFR*-mt LA was significantly lower than that in LA with wild-type *EGFR* (*EGFR*-wt). We demonstrated that the expression of CXCL9, 10 and 11, which comprises a gene cluster on chromosome 4, was significantly downregulated in *EGFR*-mt LA cell lines and that oncogenic EGFR signaling inhibits histone H3 acetylation near the gene cluster through the activation of histone deacetylases (HDACs), suggesting the intrinsic CXCL chemokine suppression in LA cells through an epigenetic mechanism under EGFR signaling. This study could explain one of the mechanisms of ICI resistance in *EGFR*-mt LA, which may contribute to the development of therapeutic strategies to mitigate ICI resistance in *EGFR*-mt LA.

## Materials and methods

### The Cancer Genome Atlas (TCGA) data analysis

The mRNA levels of *CD8A* were comparatively analyzed according to the mutational status of *EGFR*, *ALK*, and *KRAS* in LA (n = 230) cases curated in TCGA database (TCGA, Nature 2014). The mRNA levels of chemokines and chemokine receptors whose mRNA levels were positively correlated with those of *CD8A* were searched in the same dataset. All analyses were conducted using cBioPortal (http://www.cbioportal.org/)^14,15^.

### Immunohistochemical (IHC) analysis

The surgical specimens of patients with LA with or without known *EGFR* mutations (mutant, n = 56; wild-type, n = 84) were subjected to IHC using anti-CD8a mouse mAb (1:1,000; Proteintech, 66868-1-Ig). IHC staining was performed using Ventana Discovery XT (Roche, Japan) following the manufacturer’s instructions. The IHC images were captured using a virtual slide scanner, NanoZoomer-SQ C13140 (Hamamatsu, Japan). The CD8a-positive spots were automatically counted in three areas (200 × 200 µm^2^) of each sample using cellSens Dimension (Olympus, Japan).

### Cell lines

HCC827, NCI-H1373, A549 (purchased from American Type Culture Collection) and PC-9 (purchased from RIKEN Cell Bank) cells were cultured in Rosewell Park Memorial Institute (RPMI)-1640 (WAKO, Japan) medium supplemented with 10% fetal calf serum and penicillin/streptomycin at 37 °C and 5% CO_2_. All cell lines were used immediately after purchase (HCC827 and PC-9; *EGFR-*mt (Exon19 deletion)) or after authentication using short tandem repeat DNA typing (NCI-H1373 and A549; *EGFR-*wt LA).

### Multiplex chemokine assay

HCC827 or PC-9 cells were seeded at 1 × 10^6^ cells in a 60 mm dish and cultured overnight. The culture medium was replaced with 3 mL of fresh complete medium, and the cells were incubated with DMSO or AZD9291 (100 nM) (ChemScene,CS-2018) for 24 h in biological triplicates. In some experiments, the cells were stimulated with 10 ng/mL IFN-γ (PeproTech, #300-02) at 6 h post-DMSO/AZD9291 treatment. The chemokine concentrations in the culture supernatant were determined using the Bio-Plex suspension array system and Bio-Plex Pro human chemokine assays (Bio-Rad, 171AK99MR2 Japan), following the manufacturer’s instructions. The quantification was performed using biological triplicates.

### Reverse transcription quantitative polymerase chain reaction (RT-qPCR)

The methods of RNA extraction, cDNA synthesis, and RT-qPCR were described previously^16^. The relative mRNA expression levels of *CXCL9, 10, 11, HDAC1, 2, 3,* and* 4* in the cell lines were determined using the ΔΔCt method. The mRNA levels of the target genes were normalized to those of *ACTB* or *18S rRNA*, as well as those in DMSO-treated cells, as a reference. Relative quantification (RQ) was calculated as (test sample mRNA level normalized by endogenous control) / (reference sample mRNA level normalized by endogenous control). Quantification was performed using biological triplicates. Primer sequences are listed in Table 1.

**Table 1:** List of SYBR green primers.

Primer name	Sequence (5′ to 3′)
CXCL9_Fwd primer	GCAAGGAACCCCAGTAGTGAGA
CXCL9_Rev primer	TAGTCCCTTGGTTGGTGCTGAT
CXCL10_Fwd primer	TTCCTGCAAGCCAATTTTGTC
CXCL10_Rev primer	TCTTCTCACCCTTCTTTTTCATTGT
CXCL11_Fwd primer	CGAAGCAAGCAAGGCTTATAATC
CXCL11_Rev primer	AGATGCTCTTTTCCAGGACTTCATA
HDAC1_Fwd primer	GGACGAAGACGACCCTGACA
HDAC1_Rev primer	CCTCACAGGCAATTCGTTTGT
HDAC2_Fwd primer	TCAAGGAGGCGGCAAAAA
HDAC2_Rev primer	GGGTCATGCGGATTCTATGAG
HDAC3_Fwd primer	GCCTTCAACGTAGGCGATGA
HDAC3_Rev primer	TAACGCGAGCAGAACTCAAAGA
HDAC4_Fwd primer	AGCAATGAGCTCCCAAAG
HDAC4_Rev primer	CCAGAAAGTCCATCTGGAT
ACTB_Fwd primer	TGGATCAGCAAGCAGGAGTATG
ACTB_Rev primer	GCATTTGCGGTGGACGAT
18S rRNA_Fwd primer	CGAACGTCTGCCCTATCAACTT
18S rRNA_Rev primer	ACCCGTGGTCACCATGGTA

### Immunoblotting analysis of nuclear extracts

*EGFR*-mt and *EGFR*-wt LA cells were treated with DMSO (Control) or AZD9291 (1 µM) for 24 h, then the nuclear proteins were extracted using Nuclear Extraction Kit (Active Motif, 40010) in biological triplicates. The protein concentrations were measured using DC Protein Assay Kit (Promega, 5000112JA). The methods of SDS-PAE, and immunoblotting were described previously^16^. The primary (anti-histone H3ac (pan-acetyl) Ab (Active Motif, 39040), anti-Lamin A/C Ab (Active Motif, 39287)) and secondary (goat anti-rabbit IgG (GE Healthcare, NA931)) antibodies were used at a dilution of 1:1000. Immunoreactivity was detected using Fusion SoloS (M&S Instruments Inc., Japan).

### Nuclear HDAC activity measurement

The nuclear proteins were extracted as mentioned in Immunoblot analysis. The nuclear HDAC activity was measured in biological triplicates using HDAC Assay Kit (Active Motif, .56200), which is a biological assay to determine the HDAC activity using a short peptide substrate that contains an acetylated lysine residue. The HDAC activity is determined as the concentration of active HDAC by making a standard curve.

### Assay for transposase-accessible chromatin with high-throughput sequencing (ATAC-seq) analysis

HCC827 cells were treated with DMSO or AZD9291 (1 mM) in replicates for 18 h. The cells (3 × 10^5^) were then frozen in cell stock solution (Cell Banker 1, TAKARA) and transported to DNAFORM Life Science Research Center 402 (Yokohama, Japan) to perform ATAC-seq. Next-generation sequencing was performed using 150 bp pair-end sequences at 40 million reads/sample. The quality of the FASTQ sequences was determined using Fast QC. The quality-filtered sequences were mapped to the reference genome hg38 using BWA. BAM files were used for peak calls with PePr, in which the replicate peaks were merged. The detected peaks were annotated using HOMER. The motifs within the annotated peaks were searched using FIMO (http://meme-suite.org/tools/fimo). The significantly increased open chromatin peaks in the AZD9291-treated group were visualized using the IGV genomic browser.

### Cleavage under targets and tagmentation (CUT & Tag) assay

HCC827 cells were treated with DMSO (control) or AZD9291 (100 nM) for 24 h. Next, the cells were collected and analyzed using the CUT&Tag-IT Assay Kit (Active Motif, 53160) and histone H3K27ac antibody (Active Motif, 39034), following the manufacturer’s instructions. Genomic libraries containing fragments enriched by binding to anti-histone H3K27ac Ab were sequenced using an Illumina NovaSeq 6000 (Illumina, Inc.). The fastq files were uploaded to Basepair (https://activemotif.basepairtech.com/) and the differences in histone H3K27ac peaks between DMSO and AZD9291 treated samples were searched.

### Statistical analysis

Student’s t-test was performed using IBM SPSS Statistics 25.0.0. Statistical significance was set at p < 0.05.

### Ethics approval and consent to participate

The study using surgical specimens was approved by the Ethics Committee of Shiga University of Medical Science, and conducted in accordance with the Declaration of Helsinki. Informed consent was obtained from all patients.

## Results

### Infiltration of CD8^+^ T cells to tumor microenvironment (TME) is downregulated in EGFR-mt LA

**Figure 1:**
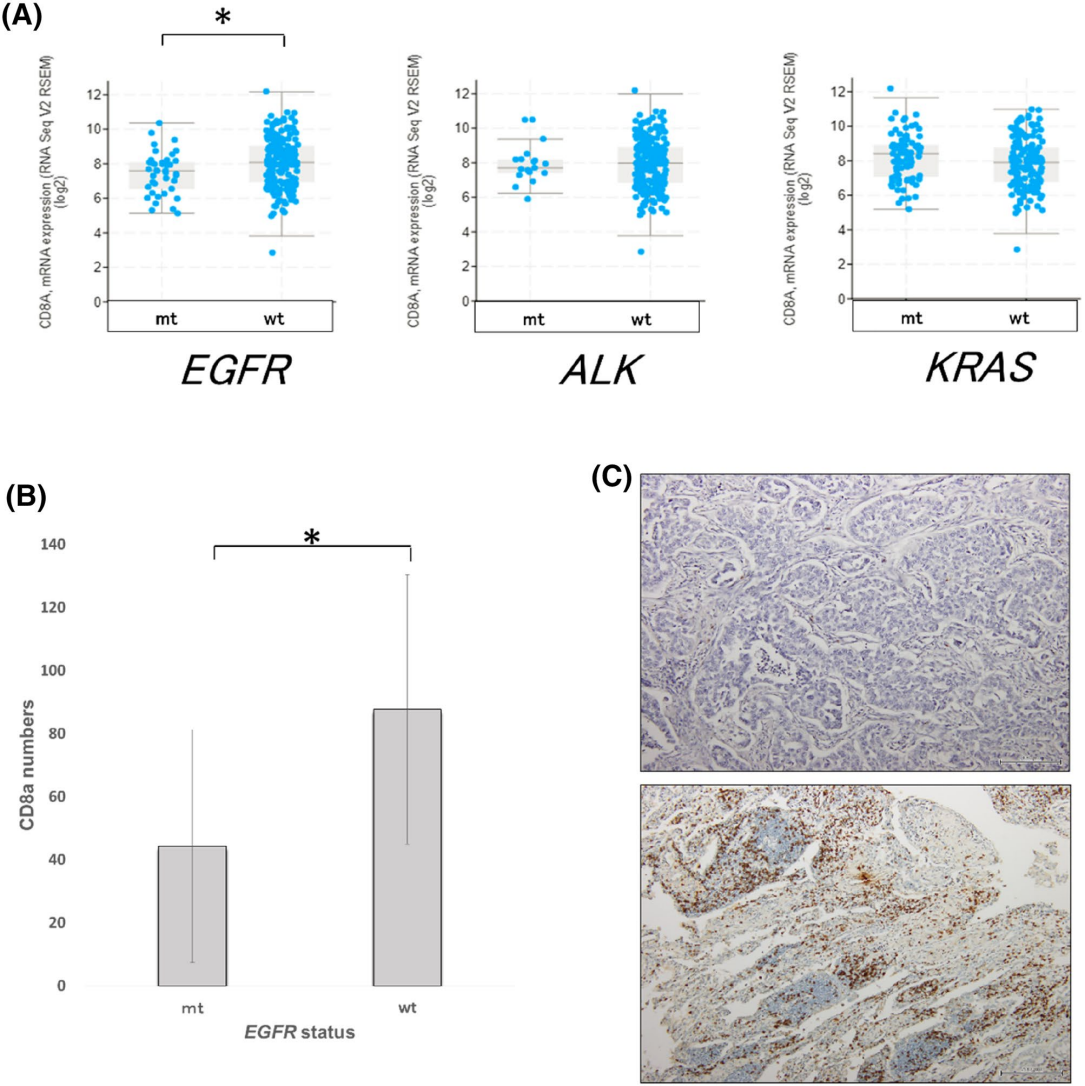
EGFR-mutated (*EGFR*-mt) lung adenocarcinoma (LA) exhibits a lower number of CD8^+^ T cells than LA with wild-type EGFR (*EGFR*-wt) LA. (**A**) Comparative analysis of the *CD8A* mRNA levels in human LA (TCGA, Nature 2014) according to the mutational status of *EGFR*, *ALK*, and *KRAS*. *EGFR* mt (n = 40; 17%), *ALK*-mt (n = 19; 8%), and *KRAS*-mt (n = 83; 36%). *p < 0.01. (**B**) Comparison of the CD8a-positive cells between *EGFR*-mt (n = 56) LA and *EGFR*-wt (n = 84) LA using immunohistochemical analysis (IHC). Mean CD8a-positive cells in three 200 × 200 µm^2^ areas were calculated for each sample. Vertical bars indicate the mean CD8a cells and the error bars indicate the standard deviation. *p < 0.0001. (**C**) Representative IHC images. Upper and lower panels indicate *EGFR*-mt and *EGFR*-wt LA, respectively. Horizontal scale bar: 200 µm.

As several previous reports indicated that CD8^+^ T cell infiltration was reduced in *EGFR*-mt LA^10,17,18^, we tried to confirm this phenomenon. In TCGA dataset, the mRNA levels of *CD8A* were significantly lower in *EGFR*-mt than *EGFR*-wt LA (TCGA, Nature 2014) (p < 0.01). However, the mRNA levels of *CD8A* did not significantly vary according to the mutational status of *ALK* or *KRAS* in the same dataset (Fig. 1A). The detailed mutational status of these driver genes is listed on Table S1. IHC of CD8a using surgical specimens confirmed that the number of CD8a^+^ T cells were significantly lower in *EGFR*-mt than *EGFR*-wt LA. The number of CD8a-positive cells in LA with *EGFR* mutation (exon 18 G719 missense mutation, exon 19 deletion, and exon 21 (L858R) except exon 20 Ins (n = 1) were significantly lower than *EGFR*-wt LA (p < 0.05). (Table S2). This indicated that *EGFR*-mt LA was associated with a decreased infiltration of CD8^+^ T cells into the TME (Fig. 1B,C).

Next, the correlation between the mRNA levels of *CD8A* and various chemokines or chemokine receptors was examined in the TCGA LA dataset (TCGA, Nature 2014) to speculate the chemokines possibly involved in CD8^+^ T cell recruitment to the TME (Table 2). Several chemokines and their receptors were significantly and positively correlated with the mRNA levels of *CD8A* (e.g., *CXCL9*, *CXCL10*, *CXCL11*, *CXCR3*, *CCL4*, *CCL5*, and *CCR5*). These chemokines may contribute to CD8^+^ T cell recruitment in the human LA TME.

**Table 2:** Chemokines and chemokine receptors, whose mRNA levels were significantly correlated to *CD8A* mRNA level in TCGA data set.

Gene	Speaman’s correlation	p-value	q-value
Chemokines			
* CCL5*	0.906	3.61e−87	3.61e−83
* CXCL9*	0.811	7.02e−55	7.03e−52
* XCL2*	0.746	3.89e−42	1.36e−39
* CCL4*	0.740	3.77e−41	1.24e−38
* CXCL11*	0.724	1.39e−38	3.75e−36
* CXCL10*	0.721	2.98e−38	7.95e−36
* CXCL13*	0.649	7.75e−29	1.15e−26
* XCL1*	0.608	1.20e−24	1.37e−22
* CCL19*	0.566	6.62e−21	5.81e−19
* CCL4L1*	0.539	1.03e−18	7.33e−17
Chemokine receptors			
* CXCR6*	0.894	2.37e−81	1.19e−77
* CXCR2P1*	0.734	3.55e−40	1.11e−37
* CCR5*	0.755	1.02e−43	3.99e−41
* CXCR3*	0.719	7.21e−38	1.85e−35
* CXCR4*	0.584	2.18e−22	2.14e−20
* CCR8*	0.564	1.09e−20	9.22e−19
* CXCR5*	0.562	1.41e−20	1.16e−18
* CCR2*	0.553	7.91e−20	6.09e−18
* CCR4*	0.539	9.95e−19	7.08e−17
* CCR6*	0.432	6.95e−12	2.56e−10

Top 10 chemokines and chemokine receptors showing positive correlation to *CD8A* mRNA level in Lung Adenocarcinoma data set (TCGA, Nature 2014) are presented according to the order of Spearman’s Correlation.

### EGFR signaling suppressed the production of several chemokines related to CD8^+^ T cell recruitment

**Figure 2:**
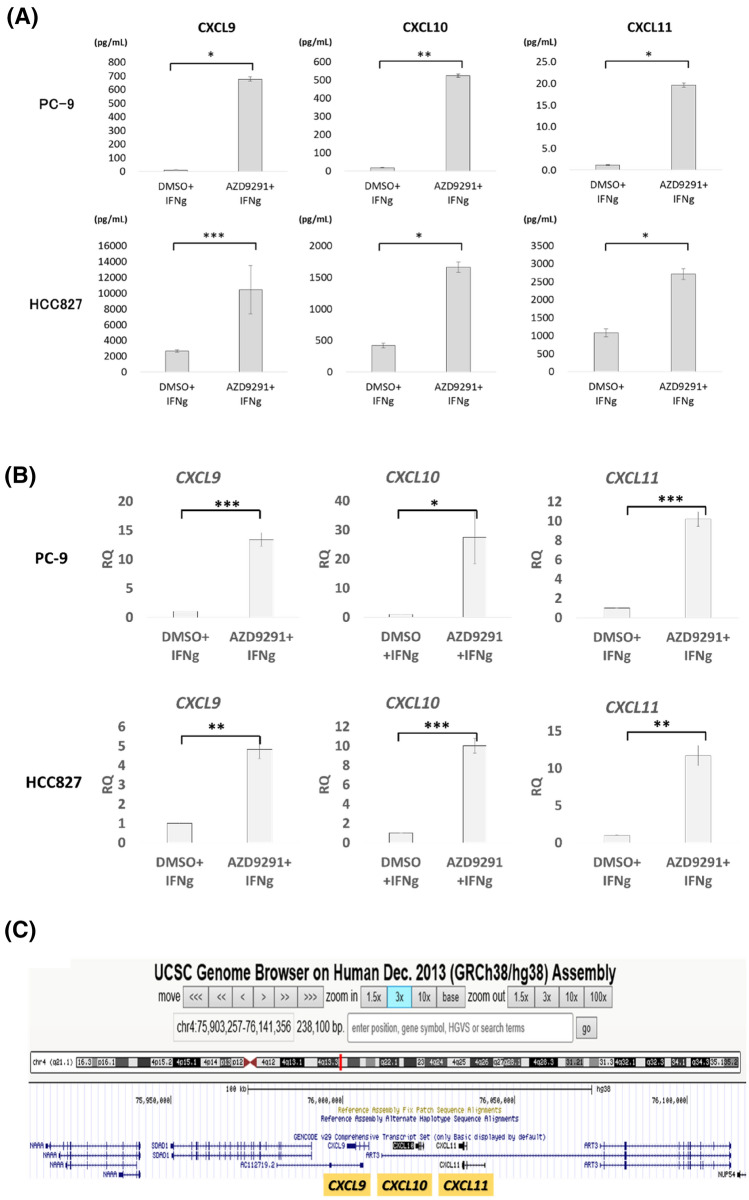
EGFR signaling suppresses chemokine expression in *EGFR*-mutated (*EGFR*-mt) lung adenocarcinoma (LA). (**A**) The levels of CXCL9, CXCL10, and CXCL11 in the culture supernatant of PC-9 and HCC827 LA cells were quantified using the Bio-Plex suspension array system after treatment with dimethyl sulfoxide (DMSO) or AZD9291 and stimulation with IFN-γ. Biological triplicates were used for the analysis. *p < 0.0005, **p < 0.0001, ***p < 0.05 (Student’s t-test). (**B**) RT-qPCR of *CXCL9*, *10*, and *11* of PC-9 and HCC827 LA cells treated with DMSO or AZD9291 and INF-γ stimulation. *p < 0.05, **p < 0.01, ***p < 0.005. Biological triplicates. (**C**) Mapping of *CXCL9, CXCL10* and *CXCL11* genes on chromosome 4 on UCSC genome browser (http://genome.ucsc.edu). The 3 *CXCL* chemokines constitute a gene cluster of 55 kb. The release date is 2018/04/11.

Since some oncogenic signaling pathways, such as Wnt/β-catenin signaling, suppress tumor-derived chemokine expression in melanoma, which results in the reduction of tumor-infiltrating T cells (TILs)^19^, we speculated that EGFR signaling might also suppress some intrinsic chemokine production in human LA cells. Treatment with EGFR-TKI, AZD9291, significantly upregulated the expression levels of CXCL9, 10, and 11 in two *EGFR*-mt LA cell lines, PC-9 and HCC827 cells (exon 19 deleted mutation) at both protein and mRNA levels (Fig. 2A,B). We also observed the increase of CXCL10 in other *EGFR*-mt LA cell lines, NCI-H1975 and II-18 (L858R missense mutation), with lesser degree than exon 19 deleted LA cells (Fig. S1A,B). We addressed whether the re-expression of the chemokine CXCL10 could increase T cell migration. We conducted in vitro CD8^+^ T cell migration assay using the culture supernatant of HCC827 cells (*EGFR-*mt) with or without AZD9291 ± anti-CXCL10 mAb. AZD9291 increased T cell migration significantly, and this increase was blocked with anti-CXCL10 mAb, suggesting re-expression of CXCL10 could increase T cell migration (Fig.S1C). We also found focal CXCL10 protein expression in *EGFR*-wt, but not in *EGFR*-mt LA cells by IHC (Fig. S2). We noticed that *CXCL9*, *10* and *11* and their corresponding receptor, *CXCR3,* were included in the list of Table 2. As we stated in Introduction, these chemokines/chemokine receptor are important for Th1 immune cell recruitment/response and anti-tumor effect of anti-PD1 Ab^12,13^. Therefore, we speculated oncogenic EGFR signal-driven suppression of these chemokines may have important roles in the reduced TILs and refractoriness of anti-PD1/PD-L1 Ab, and tried to elucidate the mechanism.

### EGFR signaling suppresses open chromatin formation around the CXCL9, 10, and 11 gene loci in EGFR-mt LA cells

We conducted promoter analyses of *CXCL10* using a luciferease vector ligated with the 0.4 and 1.2 kb DNA sequence of *CXCL10* upstream from transcription start site (TSS), by focusing on IFN regulatory factor-1 (IRF-1) because an IRF-1 binding site, IFN-stimulated response element (ISRE), is located at the 5’-proximal sequence of *CXCL10* (Fig. S3A), and previous reports indicated that EGFR signal downregulates IRF-1 and CXCL10^10,20^. However, oncogenic EGFR signal did not suppress the IRF-1 promoter activity based on our luciferase promoter assays (Fig. S3B–D). Therefore, we speculated that EGFR signal regulates the chemokine expression by suppressing the regulatory regions outside their promoters.

**Figure 3:**
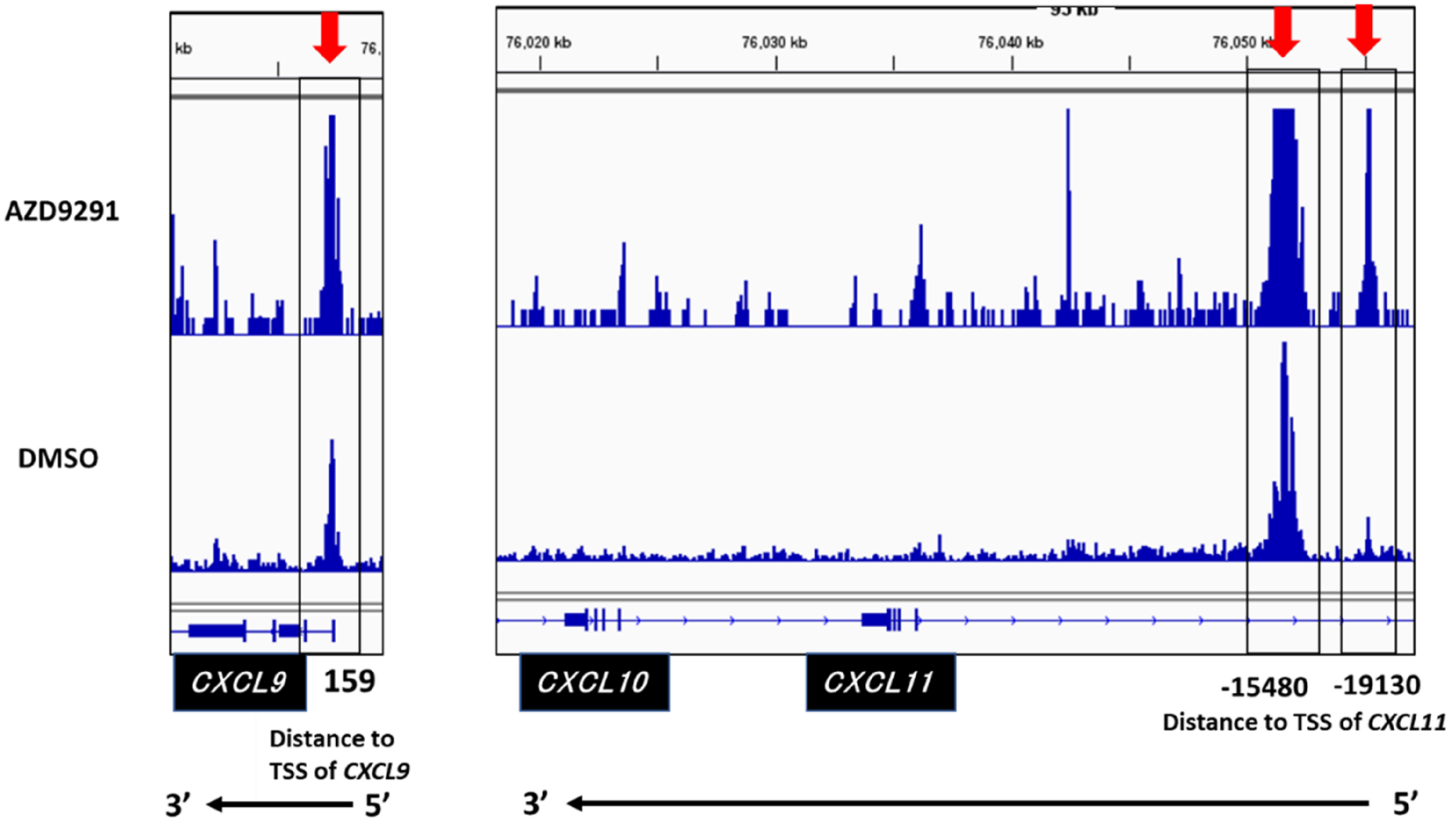
Assay for transposase-accessible chromatin using high-throughput sequencing (ATAC-seq) analysis of the sequences near *CXCL9*, *CXCL10*, and *CXCL11* in AZD9291-treated EGFR-mutated (*EGFR-*mt) lung adenocarcinoma (LA) cells. Treatment with AZD9291 generated three open chromatin peaks. One is in the first intron of *CXCL9* (+ 159 bp from the transcription start site (TSS)), while the other two are in the 5′ region of *CXCL11* (− 15,480 and − 19,130 bp from the TSS) (arrows and rectangle boxes). The large peak at the 15 kb region upstream of *CXCL11* comprises three merged peaks.

Since the expression of *CXCL9*, *10*, and *11* was simultaneously upregulated upon treatment with AZD9291 (Fig. 2A,B), and these three genes make a cluster within a region of approximately 55 kb on chr4 (75,980,790–76,036,070 bp) (Fig. 2C), we hypothesized that these three genes may be simultaneously regulated from common enhancer regions, which are not located near the proximal regions of these genes. To verify this hypothesis, *EGFR-*mt LA cells treated with AZD9291 were subjected to ATAC-seq to determine the presence of open chromatin peaks around these three genes. One region in the first intron of *CXCL9*, and two regions at a distance of 15 and 19 kb from the TSS of *CXCL11* were more open following EGFR-TKI treatment (Fig. 3). The large 15 kb peak comprised three merged peaks and no major peak was detected near *CXCL10*. The potential transcription factor (TF)-binding motifs in these loci were identified using FIMO (http://meme-suite.org/tools/fimo) (Table S3). We speculated that common enhancer regions within these peaks may contact to each promoter of these three genes simultaneously as a mechanism of a topologically associating domain (TAD)^21^, which is a DNA region whose DNA sequences contact each other, and that the common enhancer regions might be suppressed under oncogenic EGFR signaling.

### Histone deacetylase (HDAC) inhibitor increased CXCL9, 10, and 11 gene expression, and EGFR-TKI decreased HDAC1,2,3 and 4 as well as nuclear HDAC activity and increased histone H3 acetylation of EGFR-mt LA cells

**Figure 4:**
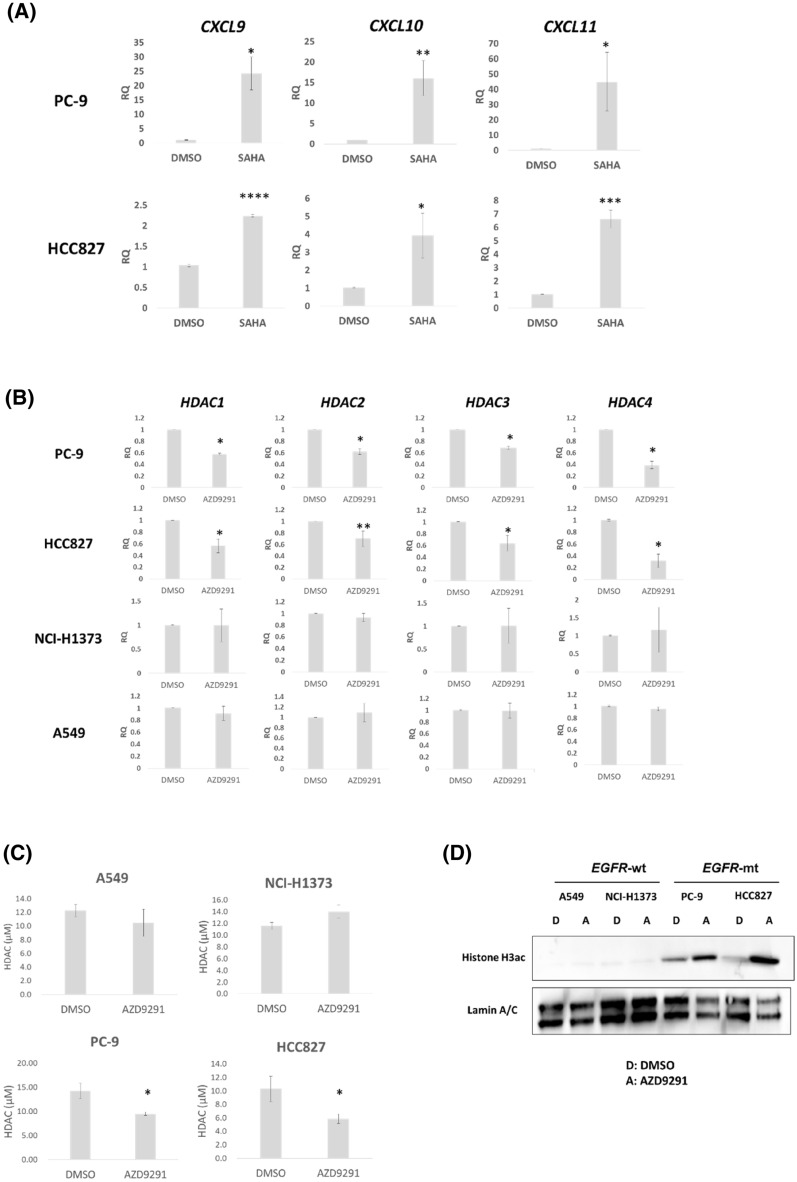
Oncogenic EGFR signaling causes induction of *HDAC1*, *HDAC2*, *HDAC3*, and *HDAC4*, associated with increased nuclear HDAC activity, nuclear histone H3 deacetylation, and chemokine suppression in *EGFR*-mt LA cells. (**A**) RT-qPCR of *CXCL9*, *CXCL10*, and *CXCL11* with or without 10 μM SAHA (vorinostat) in two *EGFR*-mt LA cells. *p < 0.05, **p < 0.005, ***p < 0.0005, ****p < 0.00005. (**B**) RT-qPCR of *HDAC1*, *HDAC2*, *HDAC3*, and *HDAC4* with or without AZD9291 in two *EGFR*-mt and two *EGFR*-wt LA cells. *p < 0.01, **p < 0.05. (**C**) Nuclear HDAC activity with or without AZD9291 in the two *EGFR*-mt and two *EGFR*-wt LA cells. *p < 0.05. (**D**) Immunoblot of histone H3 acetylation of the nuclear extract with or without AZD9291 in the two *EGFR*-mt and two *EGFR*-wt LA cells. LaminA/C is a loading control. All the experiments were performed in biological triplicates.

*EGFR*-mt LA cells (PC-9 and HCC827) treated with the pan-HDAC inhibitor, vorinostat (Active Motif, 14027), showed a significant increase in *CXCL9*, *10*, and *11* mRNA expression (Fig. 4A). However, the effects of vorinostat on the expression of these chemokines were also found in *EGFR*-wt LA cells (Fig. S4). *EGFR*-wt LA cells may have other activated signaling pathways, which might induce other HDACs leading to the epigenetic silencing of these chemokines. Therefore, we determined which HDACs were specifically regulated by oncogenic EGFR signaling. RT-qPCR of *HDAC1, 2*,* 3*, *4*,* 5*,* 6*,* 7*,* 8*, *9*, *10*, and *11* revealed that *HDAC1, 2*,* 3*, and* 4* mRNA levels were significantly decreased by AZD9291 in two *EGFR*-mt, but not in two *EGFR*-wt LA cells (Fig. 4B), while the remaining HDACs did not show consistent results in the two *EGFR*-mt LA cells (Fig. S5). This suggests that oncogenic EGFR signaling specifically activates the expression of *HDAC1, 2*,* 3*, and *4*.

Furthermore, nuclear HDAC activity was significantly suppressed by AZD9291 compared to DMSO in the two *EGFR*-mt, but not in the two *EGFR*-wt LA cell lines (Fig. 4C). Additionally, nuclear histone H3 acetylation increased with AZD9291 treatment in the two *EGFR*-mt, but not in the two *EGFR*-wt LA cells (Fig. 4D). These results suggest that oncogenic EGFR signaling increases HDAC activity through the induction of HDAC1, 2, 3, and 4, resulting in the suppression of histone acetylation in enhancer regions, which could cause epigenetic silencing.

### Oncogenic EGFR signaling suppressed histone H3K27 acetylation at one of the ATAC-seq open chromatin peaks

**Figure 5:**
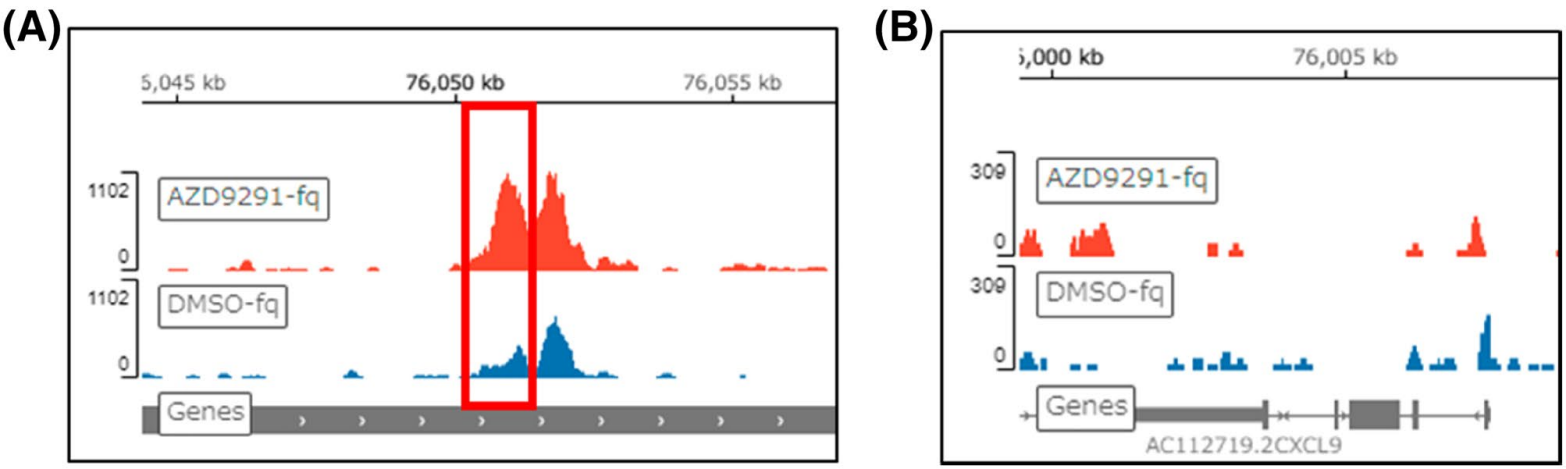
Oncogenic EGFR signaling suppressed histone H3K27 acetylation at 15 kb upstream of *CXCL11* TSS, which corresponded to the open chromatin peak by ATAC-seq. Histone H3K27 acetylation peaks are represented in Integrative genomic view (IGV). (**A**) Red histogram (AZD9291-treated) showed a significantly higher peak than blue histogram (DMSO-treated) at chr4:76,050,566–76,051,369 (p = 10^–31^, q = 10^–25^) (red rectangle), which overlapped to the open chromatin peak by ATAC-seq at chr4: 76,051,000–76,052,100. (**B**) Histone H3K27 acetylation peak in the 1st intron of *CXCL9* (chr4: 76,007,200–76,007,500) did not show significant difference between AZD9291 and DMSO.

To confirm whether oncogenic EGFR signaling suppressed histone H3 acetylation at the same open chromatin peaks identified by ATAC-seq, we conducted a CUT&Tag assay to identify histone H3K27 acetylation peaks after AZD9291 treatment. Compared to the control DMSO treatment, a significant increase in histone H3K27 acetylation was observed at chr4:76,050,566–76,051,369, which overlapped with one of the ATAC-seq open chromatin peaks at chr4_76,051,000_76,052,100, at 15 kb upstream of *the CXCL11* TSS (Fig. 5A). The other two peaks at the 1st intron of *CXCL9* and 19 kb upstream of *CXCL11* TSS were not significantly different in the CUT&Tag assay (Fig. 5A,B). Because we used anti-histone H3K27 mAb as a validated Ab for CUT&Tag assay, other histone H3 or histone H4 acetylation could not be evaluated. These results suggest that oncogenic EGFR signaling suppresses histone H3K27 acetylation at the remote region of these three chemokine genes, making the chromatin conformation an inactive, closed form, which might result in the suppression of chemokine transcription.

## Discussion

Oncogene addiction plays a role, not only in tumorigenesis or tumor progression, but also in immune evasion in mouse and human tumors^22,23^. Recently, oncogenic pathway-mediated non-T cell inflamed TME has been identified as one of the causes for the resistance of cancers to ICIs^24^. For example, oncogenic Wnt/β-catenin signaling in melanoma decreased T cell infiltration by downregulating the expression of CCL4, which recruits the basic leucine zipper TF ATF-like 3 (BATF3)-lineage dendritic cells (DCs) that cross-present tumor-derived antigens through major histocompatibility complex class I^19^. Other gain-of-function (*myc*)^25^, or loss-of-function (*LKB1*, *PTEN*, and *TP53*)^26–29^ mutations also resulted in a decreased T cell infiltration in the TME. Based on these previous observations, we speculated that oncogenic EGFR signaling might also contribute to the non-T cell inflamed TME, as most of the *EGFR*-mt LA cases exhibited higher resistance to anti-PD1 and anti-PD-L1 mAb than *EGFR*-wt LA cases^5–7^. In this study, the mRNA level of *CD8A* and the infiltration of CD8^+^ T cells were confirmed to be lower in *EGFR*-mt than *EGFR*-wt LA in TCGA dataset and surgical specimens, respectively (Fig. 1A,B). Hence, this study aimed to elucidate the underlying mechanism with a focus on the regulation of chemokine gene expression.

Offin et al. reported that the tumor mutation burden (TMB) of *EGFR-*mt LA was significantly lower than that of *EGFR-*wt LA^9^. TMB is one of the predictive factors of ICI efficacy^30,31^. Hence, the low TMB in *EGFR-*mt LA can potentially explain the resistance to immunotherapy. However, a low TMB is not directly associated with the decreased number of CD8^+^ T cells in the TME, which exhibits suppressed T cell infiltration in the effector phase (but not in the induction phase) of the cancer immunity cycle^32^. In the effector phase, the suppression of recruitment or proliferation of effector CD8^+^ T cells in the TME must occur. A low TMB is closely associated with the suppression of neoantigen-specific T cells in the induction phase. Therefore, this cannot explain the formation of non-T cell inflamed TME.

Similar observations to ours have recently been reported by Sugiyama et al.^10^. Several differences were found between their results and ours. At first, they reported that the mRNA expression of *FOXP3* in *EGFR*-mt was higher than that in *EGFR*-wt LA, which suggested that the number of regulatory T cells (Tregs) increased in *EGFR*-mt LA. However, the mRNA levels of *FOXP3* and chemokines recruiting Treg (*CCL22*, *CCL17*, and *CXCL12*) were not significantly different between *EGFR*-mt and *EGFR*-wt LA in TCGA dataset (Fig. S6A). Treatment with AZD9291 significantly downregulated the expression of CCL22 in the HCC827 cells, but not in PC-9 cells, in our study (Fig. S6B). Thus, here, the relationship between oncogenic EGFR signaling and Treg recruitment was not clear. Secondly, the interpretation of the regulatory mechanism of CXCL10 suppression varied between the two studies. Sugiyama et al. claimed that EGFR downregulates CXCL10 by suppressing IRF-1^10^. Previous studies^20,33^ have also suggested that IRF-1 promotes CXCL10 transcription by binding to ISRE in the *CXCL10* 5′-proximal region. In fact, we noticed that AZD9291 significantly increased IRF-1 expression at both mRNA and protein levels in *EGFR*-mt LA cells as Sugiyama et al. (Fig. S7A)*.* We also observed that site-directed mutagenesis of ISRE of the *CXCL10* promoter significantly decreased promoter activity (Fig. S7B), suggesting that IRF-1 enhances *CXCL10* transcription through ISRE. However, treatment with AZD9291 decreased IRF-1 promoter activity (Figs. S3 and S7B), irrespective of the increase in the IRF-1 protein level. These results suggest that the transcriptional activity of IRF-1 is not simply regulated by its quantity, but through other complex mechanisms, such as post-translational modification of IRF-1 or formation of a complex with other transcription factors, which depends on oncogenic EGFR signaling. We speculated that the regulatory mechanism of EGFR-driven CXCL10 suppression does not exist in the 5’-proximal promoter region of *CXCL10*. This prompted us to examine alternative regulatory mechanisms.

In this study, the expression of three IFN-γ-responsive Th1-inducing chemokines (CXCL9, 10, and 11) was similarly increased upon treatment with AZD9291 (Fig. 2A,B). These three genetic loci were localized within a small region of approximately 55 kb. These three genes were simultaneously suppressed by histone deacetylases that were induced by oncogenic EGFR signaling. Histone deacetylation occurred at a presumed TAD near the gene cluster, which corresponded to one open chromatin peak detected by ATAC-seq. These results suggest an epigenetic suppressive mechanism of chemokine genes through the suppression of histone H3 acetylation at TAD, which was mediated by oncogenic EGFR signaling. We examined whether a DNA methyltransferase inhibitor, 5-azacytidine (5-AZA), could increase the expression of *CXCL10* in *EGFR*-mt LA cells, but did decrease the expression level (Fig. S8), suggesting promoter methylation seems to be irrelevant. Two open chromatin peaks found by the ATAC-seq assay (the 1st intron of *CXCL9* and 19 kb upstream of *CXCL11*) did not correspond to those in the CUT&Tag assay. We used an anti-histone H3K27 acetylation Ab because pan-histone H3 acetylation Ab was not validated for this assay. It is possible that other histone H3 acetylation or histone H4 acetylation peaks may be formed with AZD9291 and correspond to the two ATAC-seq peaks. Although our study suggests a role for TAD in the epigenetic regulation of chemokine genes, the presence of TAD must be demonstrated by more strict assays for genome-wide conformational studies, such as the chromosome conformation capture (3C) method^34^ or Hi-C^35^ in a future study. Furthermore, it remains to be elucidated how oncogenic EGFR signaling regulates HDAC1-4 expression, and whether HDAC1-4 deacetylate histone H3 near the *CXCL9-11* gene cluster under oncogenic EGFR signaling, which warrant further experiments in future studies.

The significance of tumor intrinsic chemokine suppression in vivo remains unclear in our in vitro results. It remains to be demonstrated whether suppression of the 3 CXCL chemokines could cause T cell-deserted tumor microenvironment. However, it has been well documented that CXCL9, 10, and 11 play crucial roles in immune activation and tumor rejection in TME via its cognate receptor, CXCR3^12^, and anti-tumor effect of anti-PD-1 therapy decreases in *CXCR3* knock-out mice^13^. Enhancer of zeste homolog 2 (EZH2)-mediated histone H3K27me3 and DNMT1-mediated DNA methylation suppress the ovarian tumor production of Th1-inducing chemokine, CXCL9 and 10, and decrease TIL in TME. Inhibitors for the DNMTs could increase the TIL and improves therapeutic effects of PD-1 Ab, suggesting that epigenetic silencing of Th1-chemokine is a tumor immune evasion mechanism^36^. In view of the significance of the CXCL9, 10, and 11/CXCR3 axis in anti-PD-1 therapy, our findings may provide a new insight to the mechanism of *EGFR*-driven resistance against ICI. Further in vivo study focusing on the relationship between suppression of the 3 CXCL-chemokines and the resistance of ICIs in *EGFR*-mt LA, and whether HDAC inhibitor (HDACi) could overcome the ICI resistance are warranted in the future study.

In summary, this study suggested that the oncogenic EGFR signaling pathway suppresses intrinsic CXCL chemokine expression in *EGFR*-mt LA cells through histone deacetylation at 15 kb upstream of *CXCL11* by HDACs which are induced under EGFR signaling. This finding may make a possibility for a new therapeutic strategy, a combination of ICI and HDAC inhibitors, which might be safer than combination of ICI and EGFR-TKI which increased interstitial pneumonia^37^. To our knowledge, this is the first report demonstrating oncogene-driven epigenetic silencing of chemokine genes, which might lead to the immune evasion of cancer, as well as the resistance to ICI, which may open a new possibility to overcome the ICI resistance of *EGFR*-mt LA.

## Supplementary Information

Supplementary Information.

## Data Availability

The data generated in this study are available within the article and its supplementary data files. The datasets of ATAC-seq and CUT&Tag generated during the current study are available in the DBJJ repository, DRA014913 and DRA014923, respectively.
