# Supplementary material for: Oncogenic epidermal growth factor receptor signal-induced histone deacetylation suppresses chemokine gene expression in human lung adenocarcinoma
Source: Sci Rep. 2023 Mar 29;13:5087. doi: 10.1038/s41598-023-32177-4 (PMC10060241; doi:10.1038/s41598-023-32177-4)
Supplement: Supplementary file 1 — Supplementary Information. [file 41598_2023_32177_MOESM1_ESM.pdf]

EGFR

mutation type	n
missense	30
In-frame del	12
In-frame ins	1
frame shift del	1
nonsense	1
total	45

ALK

mutation type	n
missense	13
nonsense	2
splice site	1
fusion	3
total	19

KRAS

mutation type	n
missense	75
nonsense	1
total	76

Table. S1 Mutational status of *EGFR, ALK* and *KRAS* in TCGA data set (Adenocarcinoma TCGA, Nature 2014).

EGFR mutation type	$CD8a^+$ cell numbers (mean±SD)	n	p-value
Exon 18 (G719 missense mutation)	37.0±39.4	5	0.043
Exon 19 deletion	40.6±29.4	29	<0.0001
Exon 21 (L858R)	49.4±43.5	19	0.0017
Exon 20 Ins	6.7	1	n.a.

Table. S2 The number of CD8a-positive cells according to *EGFR*-mutation type. Mean CD8a cell number and standard deviation, number of each mutation, and statistical significance (vs *EGFR*-wt LA) in surgical specimens. n.a.; not available

Table S3. Transcription factor (TF)-binding motifs found in the open chromatin peaks by ATAC-seq.

TF motifs in the 1st intron of *CXCL9* gene（chr4: 76,007,200_76,007,500)

motif_id	motif_alt_id	start	stop	strand	score	p-value	q-value	matched_sequence
AWWWTGCTGAGTCAT	NFE2L2	17	31	-	12.81	1.52E-05	0.00239	TATTTGGTCAGTCAT
GTCATAAAAN	Cdx2	12	21	-	13.1561	2.00E-05	0.00297	GTCATAAAAG
VGCCATAAAA	Hoxd11	13	22	-	10.3486	2.23E-05	0.00369	AGTCATAAAA
AAACYKGTTWDACMRGTTTB	GRHL2	64	83	+	11.5091	4.32E-05	0.00369	TATCTGGCTAATCAAGATTC
NGYCATAAAWCH	CDX4	11	22	-	10.3218	4.83E-05	0.00741	AGTCATAAAAGC
AAACYKGTTWDACMRGTTTB	GRHL2	63	82	-	11.2303	5.32E-05	0.00369	AATCTTGATTAGCCAGATAA
TTTTATGGCM	Hoxa11	13	22	+	9.57062	5.38E-05	0.00892	TTTTATGACT
ACTTTCACTTTC	PRDM1	53	64	+	10.5988	7.72E-05	0.013	ACATTCACCTTT
AWWNTGCTGAGTCAT	Bach1	17	31	-	9.89091	8.59E-05	0.0136	TATTTGGTCAGTCAT
NCYAATAAAA	Hoxd13	13	22	-	10.5087	9.02E-05	0.0148	AGTCATAAAA
CYHATAAAAN	Hoxa13	12	21	-	9.71839	9.55E-05	0.0154	GTCATAAAAG

TF motifs at 15kb peak of the 5'-upstream of *CXCL11* gene（chr4: 76,051,000_76,052,100)

motif_id	motif_alt_id	start	stop	strand	score	p-value	q-value	matched_sequence
CTGCCWVCTTTTRTA	ZNF7	8	22	+	11.1697	5.74E-05	0.0141	ctgtcacctctcaAA
AGGTGTCA	Tbx5	9	16	-	10.2606	7.40E-05	0.0209	AGGTGACA
TGTCANYT	Tgif2	9	16	+	8.86826	7.98E-05	0.0211	tgtcacct
SSRGCAGCTGCH	Ascl2	85	96	-	10.6242	8.82E-05	0.0237	CCGGCAGCTCCC
WATGCAAATGAG	Oct_6	69	80	+	10.3663	9.65E-05	0.0243	CATGCCAATGAC
VGCTGWCAVB	Meis1	6	15	+	10.697	9.82E-05	0.0275	cactgtcacc

TF motifs at 15kb peak of the 5'-upstream of *CXCL11* gene（chr4: 76,051,000_76,052,100)

motif_id	motif_alt_id	start	stop	strand	score	p-value	q-value	matched_sequence
ATGCATWATGCATRW	OCT:OCT-short	43	57	+	16.8727	1.09E-06	0.000226	AGGAATAATGCATGT
ATGAATWATTCATGA	OCT:OCT	43	57	+	15.6272	2.04E-06	0.000403	AGGAATAATGCATGT
ATGCATWATGCATRW	OCT:OCT-short	41	55	-	13.6606	5.67E-06	0.000588	ATGCATTATTCCTCA
GRTGMTRGAGCC	ZNF415	120	131	-	14.2061	6.61E-06	0.00182	GGCGATAGAGCA
ATGAATWATTCATGA	OCT:OCT	41	55	-	13.9586	7.25E-06	0.000719	ATGCATTATTCCTCA
ATGCATAATTCA	Pit1+1bp	44	55	-	12.4393	2.06E-05	0.00388	ATGCATTATTCC
BTBRAGTGSN	Nkx2.2	136	145	+	10.6946	4.32E-05	0.0121	ctggagtgca
ATGCATAATTCA	Pit1+1bp	43	54	+	11.4335	4.43E-05	0.00418	AGGAATAATGCA
NNTGTTTATTTTGGCA	NF1:FOXA1	103	118	+	10.7515	6.14E-05	0.0148	tttgtttgttttagac
TGTTTAYTTAGC	FoxD3	105	116	+	10.7574	7.18E-05	0.0156	tgtttgttttag
AGTAAACAAAAAAGAACANA	FOXA1:AR	93	112	-	10.2303	7.51E-05	0.0141	AACAAACAAACAAAAAATTC
GYCATCMATCAT	HOXA2	88	99	-	10.8	7.57E-05	0.0172	AAAATTCATCAT
RSCACTYRAG	Nkx2.1	136	145	-	10.1576	9.19E-05	0.0259	TGCACTCCAG

TF motifs at 15kb peak of the 5'-upstream of *CXCL11* gene（chr4: 76,051,000_76,052,100)

motif_id	motif_alt_id	start	stop	strand	p-value	q-value	matched_sequence
WDNCTGGGCA	ZNF416	96	105	-	1.23E-06	0.000465	AGCCTGGGCA
WDNCTGGGCA	ZNF416	292	301	-	1.23E-06	0.000465	AGCCTGGGCA
ACATGCCCGGGCAT	p53	95	108	-	3.62E-06	0.00266	ACCAGCCTGGGCAA
GAGCCTGGTACTGWGCCTGR	ZNF322	173	192	-	3.66E-06	0.00262	CAGCCAGGTACAGTGGCTTA
AAATCACTGC	Gfi1b	321	330	+	6.95E-06	0.00534	caatctcagc
SMCAGTCWGAKGGAGGAGGC	ZSCAN22	363	382	-	9.95E-06	0.00699	GGCAGGCTGAGGCTGGAGAA
CAAGATGGCGGC	YY1	86	97	-	1.59E-05	0.0116	CAACATGGCGAA
ACATGCCCGGGCAT	p53	291	304	-	1.69E-05	0.00622	TCCAGCCTGGGCAG
YTGWCADY	Tgif1	189	196	-	2.67E-05	0.0207	TTGTCAGC
CCAGGAACAG	AR-halfsite	180	189	-	2.70E-05	0.0201	CCAGGTACAG
ATTTCCCAGVAKSCY	ZNF143|STAF	145	159	+	2.73E-05	0.0207	acctcccaaagtgct
BTBRAGTGSN	Nkx2.2	300	309	+	3.11E-05	0.0177	ctggagtgga
AACTACAATTCCCAGAATGC	GFY-Staf	139	158	+	3.84E-05	0.0286	acctcaacctcccaaagtgc
GAGCCTGGTACTGWGCCTGR	ZNF322	34	53	+	3.90E-05	0.014	cacacacgtccacagccagc
DCYAAAAATAGM	Mef2c	63	74	-	3.92E-05	0.0298	ACCAAAAATACA
VGCTGWCAVB	Meis1	188	197	+	4.02E-05	0.0304	ggcTGACAAT
TWGHWACAWTGTWDC	DMRT1	269	283	+	4.27E-05	0.0321	ttgagacagagtttc
NCTGGAATGC	TEAD	112	121	-	4.48E-05	0.0349	ACAGGAATTC
BTBRAGTGSN	Nkx2.2	305	314	+	4.57E-05	0.0177	gtggagtgca
YSTGGGTGGTCT	Gli2	99	110	+	5.50E-05	0.0379	ccaggctggtct
GGCCCCGCCCCC	Sp1	339	350	+	5.70E-05	0.0398	acctccgcccct
AGTAAACAAAAAAGAACANA	FOXA1:AR	55	74	-	5.78E-05	0.0428	ACCAAAAATACAAAAACATT
CCAAAAATAG	Mef2a	64	73	-	5.86E-05	0.0451	CCAAAAATAC
TCATGGTGYCYTWYTCCCTTC	ZNF41	329	353	+	6.37E-05	0.0452	gctcactgcaacctccgcccctgag
TAATCCCN	Pitx1	159	166	-	6.51E-05	0.051	TAATCCCA
YDGHTACAWTGTADC	DMRT6	269	283	+	6.60E-05	0.0504	ttgagacagagtttc
TTGAMCTTTG	RARa	349	358	-	6.93E-05	0.0506	TTGAACTCAG
GWAAYHTGAKMC	Six2	107	118	-	6.97E-05	0.0542	GGAATTCGAGAC
AACAGGAAGT	Ets1-dista	113	122	-	7.78E-05	0.058	CACAGGAATT
NTNATGCAAYMNNHTGMAAY	CEBP:CEBP	84	103	-	7.81E-05	0.0577	CCTGGGCAACATGGCGAAAC
GGGGGAATCCCC	NFkB-p50,p52	81	92	-	8.08E-05	0.0571	TGGCGAAACCCC
DAGGTGTBAA	Tbx6	134	143	-	8.37E-05	0.0648	GAGGTGGGAA
AACAGGAAAT	EWS:FLI1-fusion	113	122	-	9.24E-05	0.0705	CACAGGAATT
ATTTCCTGTN	EWS:ERG-fusion	113	122	+	9.36E-05	0.0707	aattcctgtg
RSCACTYRAG	Nkx2.1	300	309	-	9.73E-05	0.0757	TCCACTCCAG
NNNGCATGTCCNGACATGCC	p63	363	382	+	9.80E-05	0.0715	ttctccagcctcagcctgcc

TF motifs at 19kb peak of the 5'-upstream of *CXCL11* gene（chr4: 76,055,100_76,055,300)

motif_id	motif_alt_id	start	stop	strand	score	p-value	q-value	matched_sequence
TCATCAATCA	Pdx1	90	99	-	11.6566	3.57E-05	0.00633	CCATCACTCA
RTGATTKATRGN	PBX2	89	100	+	11.0592	4.53E-05	0.00787	ATGAGTGATGGA
GAGGTCAAAGGTCA	TR4	34	47	-	11.0602	4.64E-05	0.00718	AAGGTGAGAGGTAG
TGATKGATGR	HOXA1	90	99	+	10.8263	6.40E-05	0.0113	TGAGTGATGG
NWAACCACADNN	RUNX2	71	82	-	11.1576	8.51E-05	0.0141	ACCACCACATAC

Potential TF binding sites found from ATAC-seq peaks using FIMO. The sequences within 1 peak in the 1st intron of *CXCL9* gene, 3 peaks and 1 peak at 15kb and 19kb from 5'-upstream of *CXCL11* gene, respectively, were analyzed.

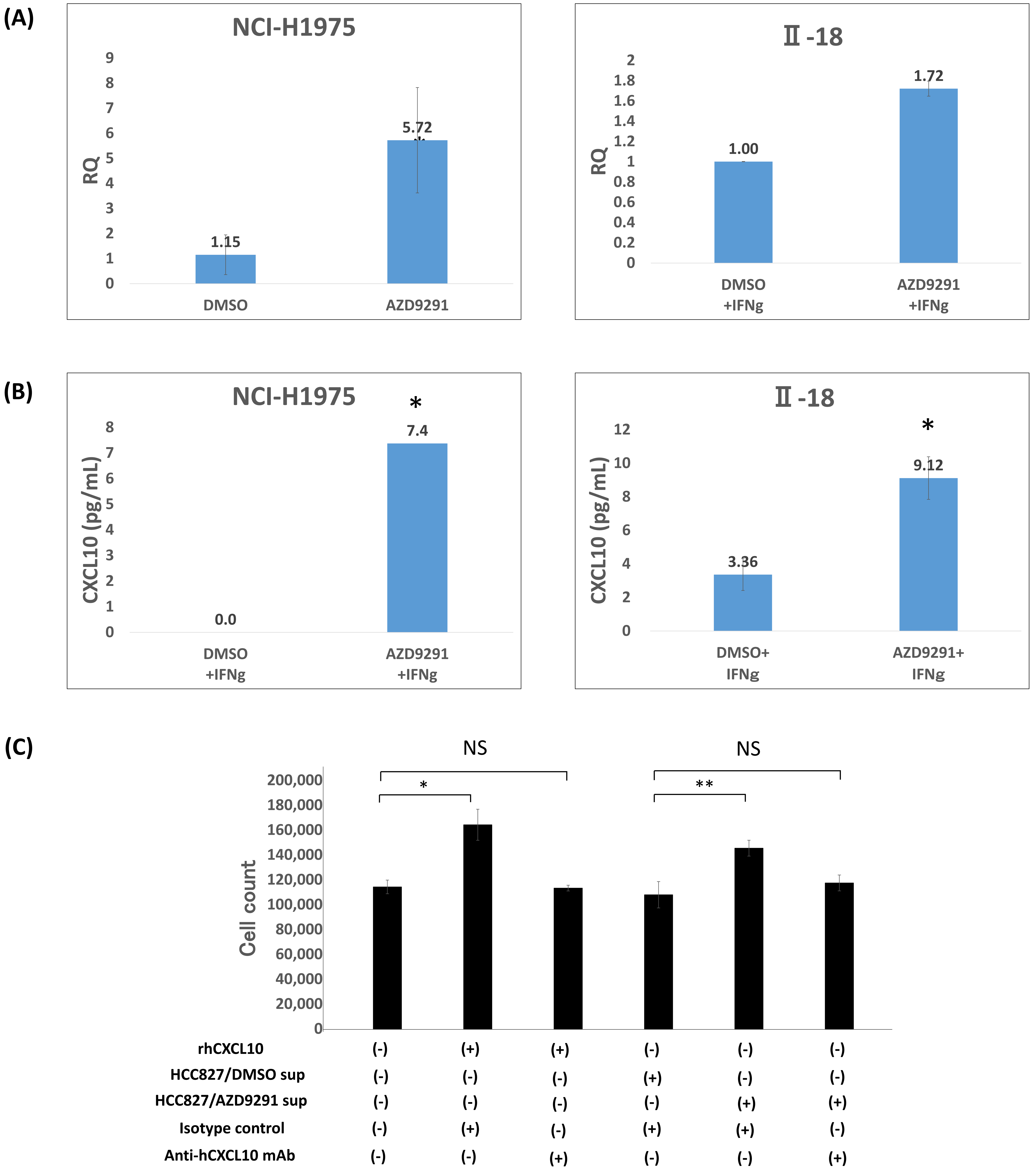

Fig. S1 The effect of AZD9291 on the CXCL10 expression level in two *EGFR*mt (L858R) LA cell lines. (A) RT-qPCR of *CXCL10* with or without AZD9291, EGFR-TKI, in NCI-H1975 (Left panel) and II-18 (Right panel). (B) ELISA of CXCL10 in the culture supernatant in two *EGFR*mt (L858R) LA cell lines. * p<0.05 (C) T cell migration assay. The number of $CD8^+$ T cells migrating into the lower wells through the Transwell inserts significantly increased depending on the presence of CXCL10 in the lower wells. rhCXCXL10 was used as a positive control. Biological triplicates were used for the analysis. *p = 0.001 and **p = 0.009 (analysis of variance). NS; not significant.

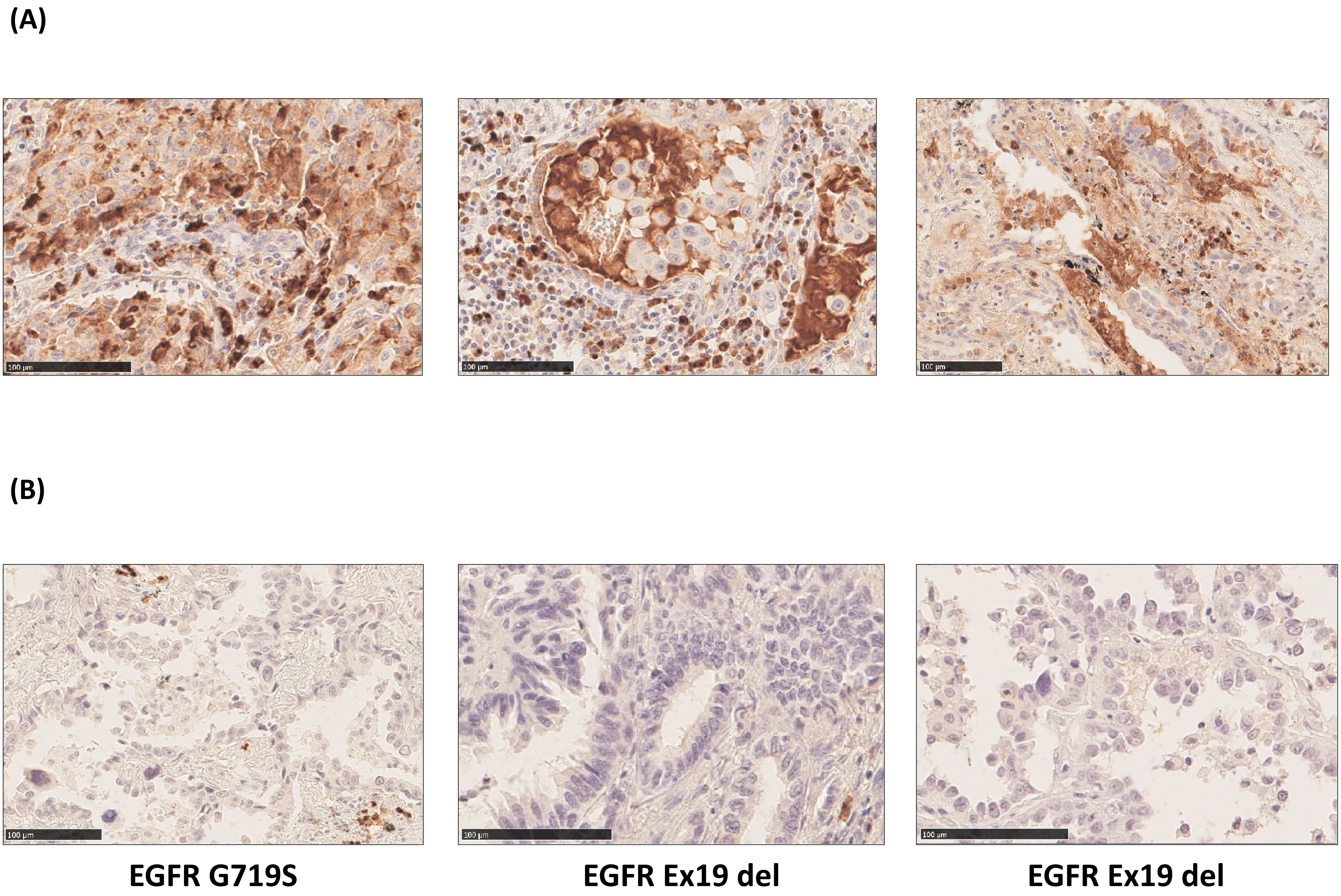

Fig. S2 Immunohistochemistry of CXCL10 in *EGFR*-mt and *EGFR*-wt LA tissues. (A) Three cases of *EGFR*-wt LA surgical specimens. Focal CXCL10-positive LA cells were found. (B) Three cases of *EGFR*-mt LA tissues. Spotty CXCL10-positive macrophages were found in the interstitium. Scale bars indicate 100μm.

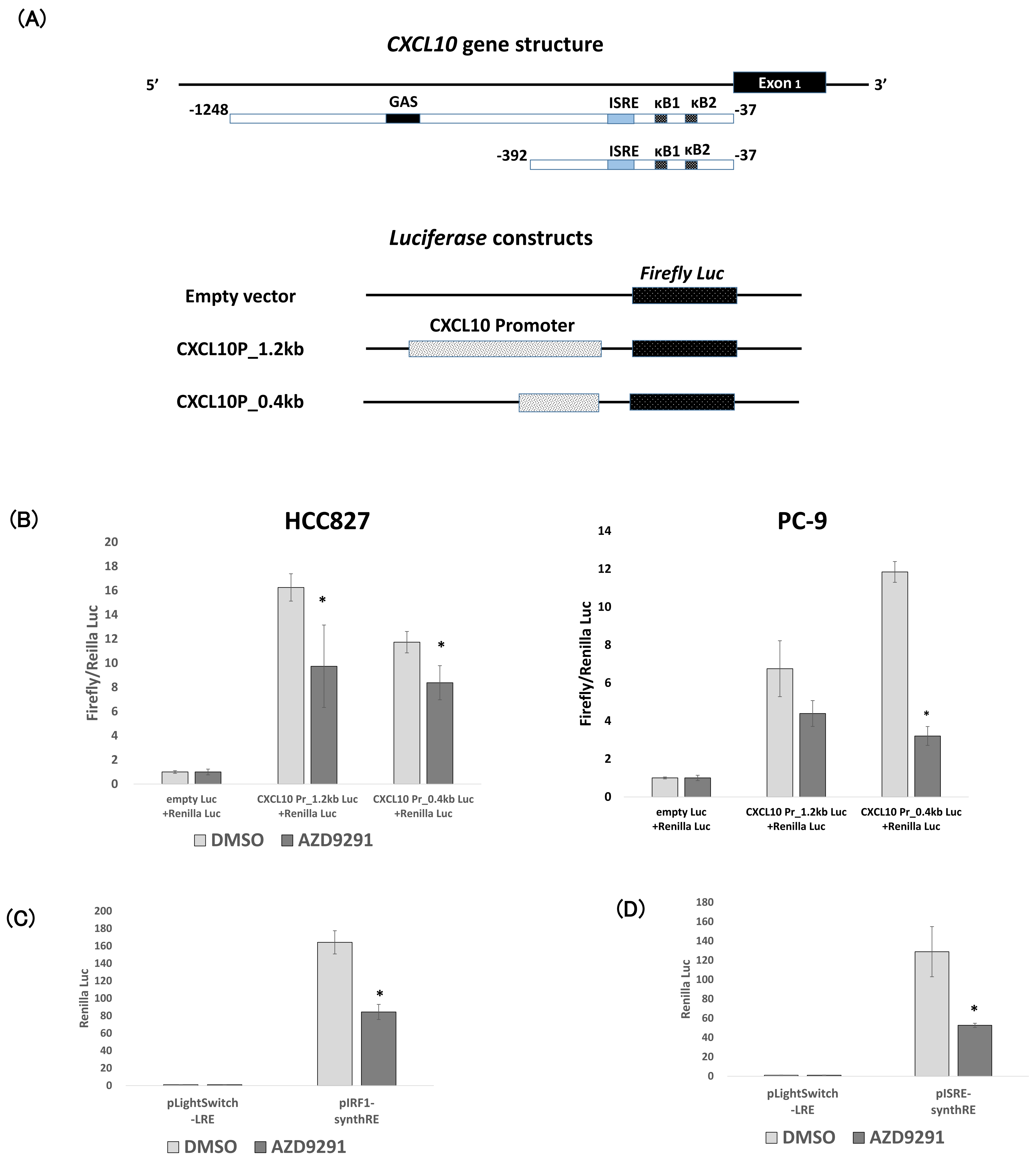

Fig. S3 ***CXCL10*** **promoter analysis.** (A) *CXCL10* gene structure and luciferase reporter plasmids. (Upper panel) A scheme of the 5'-upstream region of *CXCL10* exon 1, which was cloned using PCR. Approximate locations of GAS (−754 bp from the transcription start site (TSS)), ISRE (−215 bp from the TSS), κB1 (−170 bp from the TSS), and κB2 (−115 from the TSS). (Lower panel) A scheme of the Firefly luciferase plasmid constructs. Empty vector indicates a promoterless pGL4.17 luciferase plasmid. CXCL10P_1.2 kb and CXCL10P_0.4 kb indicate pGL4.17 plasmids containing *CXCL10* promoter sequences of different lengths (upper panel) in the 5'-upstream region of the Firefly luciferase gene. (B) *CXCL10* promoter analysis. The activity of Firefly luciferase normalized to that of Renilla luciferase activity (Firefly/Renilla luc) was compared between the DMSO-treated and the AZD9291-treated HCC827 and PC-9 cells. The baseline Firefly (empty luc)/Renilla luciferase activity varied between the DMSO-treated and the AZD9291-treated groups. Hence, the activities of different CXCL10 promoter-luciferase constructs were normalized to those of the mean empty luc/Renilla luc activity. Experiments were performed using biological quadruplicates. $*p < 0.05$ (Student's t-test compared with the DMSO-treated group). (C) IRF-1 reporter assay using synthetic IRF-1 binding site-driven Renilla luciferase vector. The activity of Renilla luciferase was normalized to that of the control plasmid (pLight Switch-LRE) without a promoter. The IRF-1 promoter activity in the AZD9291-treated HCC827 cells was significantly lower than that in the DMSO-treated HCC827 cells. Experiments were performed using biological quadruplicates. $*p < 0.005$. (D) ISRE reporter assay using synthetic ISRE-binding site-driven Renilla luciferase vector. The results of this assay were consistent with those of the IRF-1 reporter assay. Experiments were performed using biological quadruplicates. $*p < 0.05$.

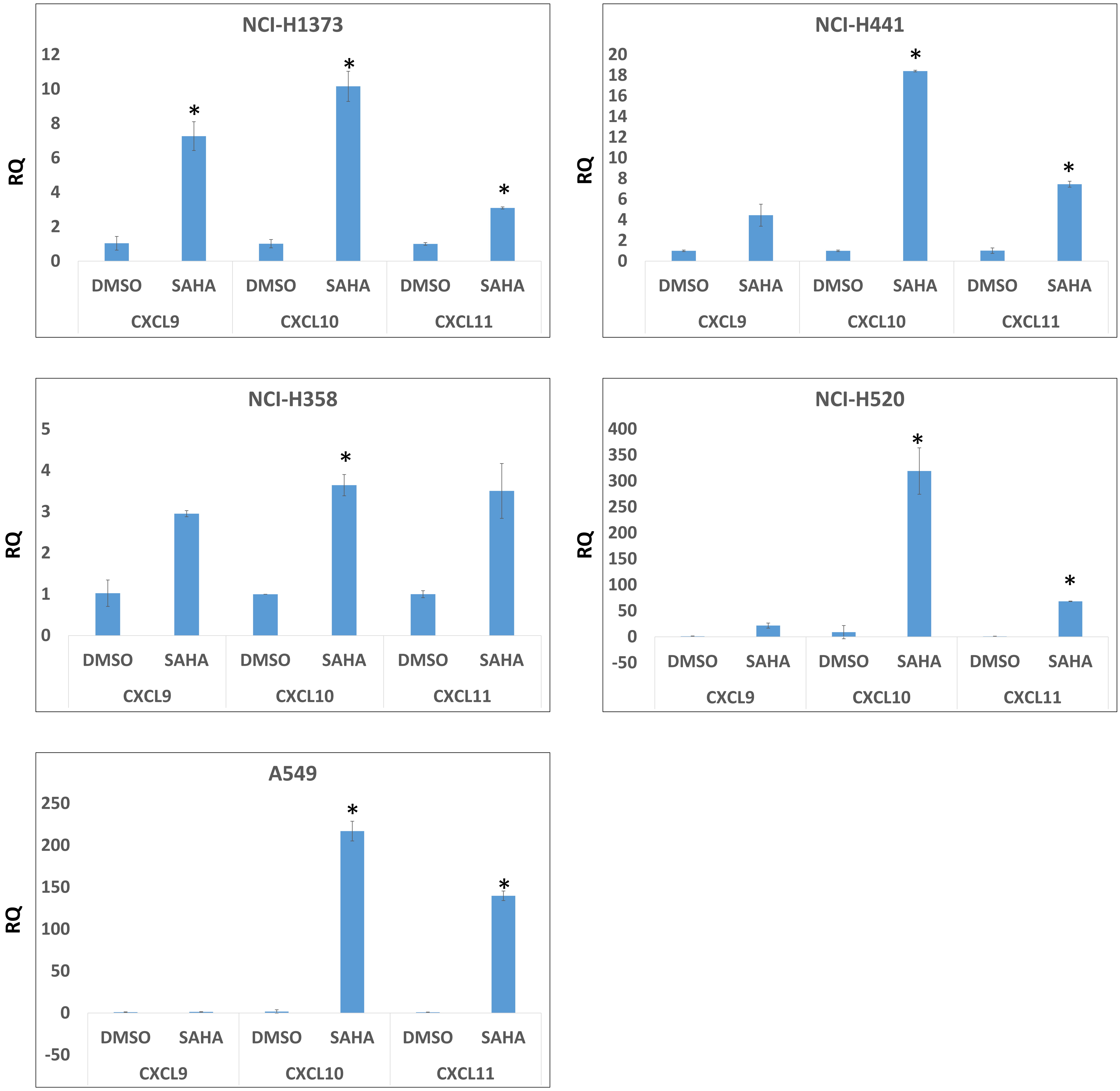

Fig. S4 The effects of vorinostat (SAHA) on *CXCL9*, *10*, and *11* mRNA levels in *EGFR*wt LA cell lines. Five *EGFR*wt LA cell lines, NCI-H1373, NCI-H441, NCI-H358, NCI-H520, and A549 were treated with DMSO or SAHA (10µM) for 24h. The extracted RNAs were applied to RT-qPCR of the chemokines using SYBR Green ΔΔCt method. RQ; relative quantitate. * $p<0.05$ (Student's t-test)

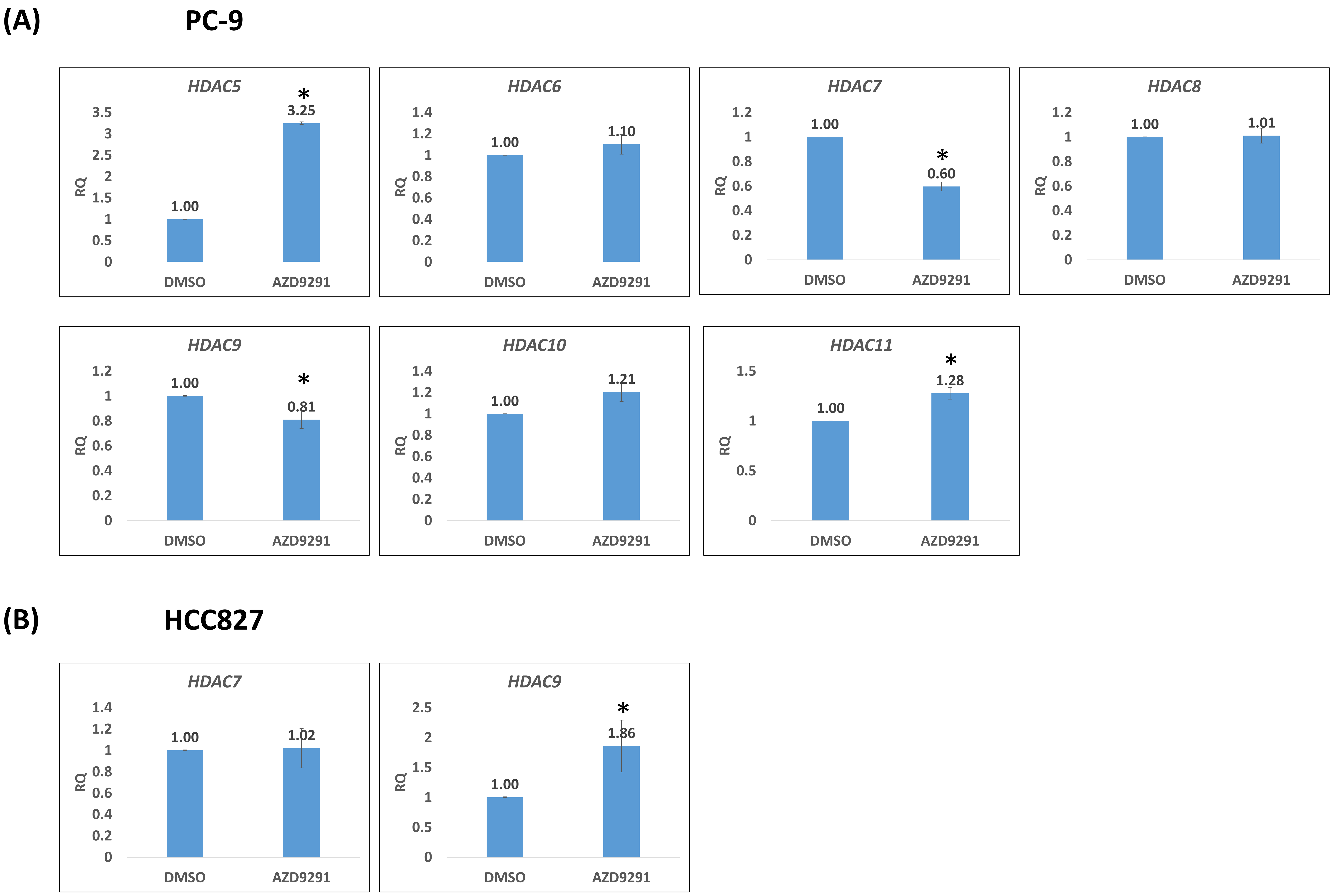

Fig. S5 The effects of EGFR-TKI (AZD9291) on *HDAC5-11* mRNA levels in two *EGFR*-mt LA cell lines. (A) PC-9. (B) HCC827. HCC827 cells were evaluated only for *HDAC7* and *HDCA9*, which were significantly decreased with AZD9291 in PC-9. Biological triplicates. RQ; relative quantitate. * $p<0.05$ (Student's t-test)

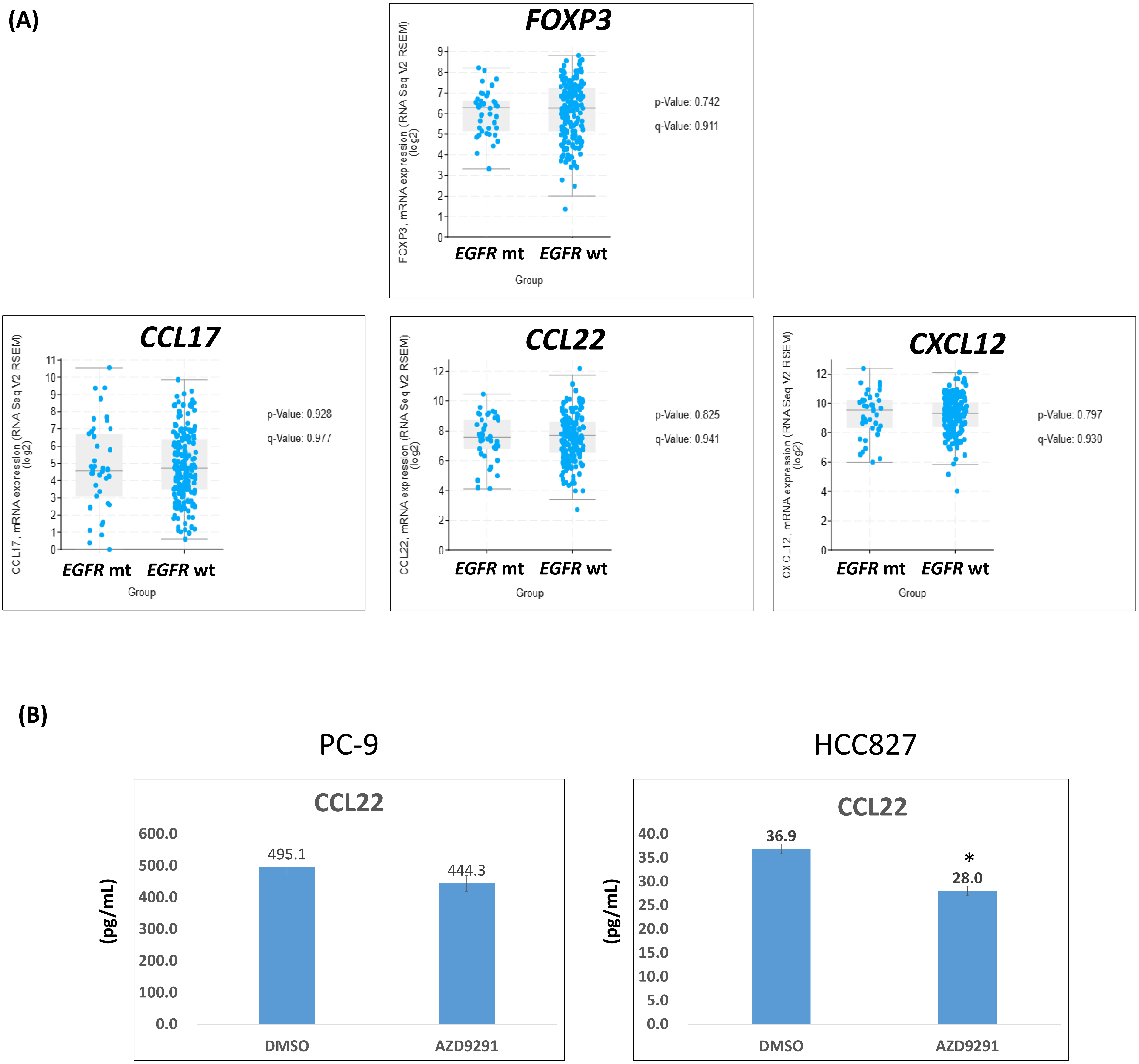

Fig. S6 The comparison of mRNA expression of *FOXP3* and Treg-recruiting chemokines between *EGFR*-mt and *EGFR*-wt lung adenocarcinoma. (A) *FOXP3* and three chemokine (*CCL22*, *CCL17*, and *CXCL12*) mRNA levels were compared between *EGFR*-mt and *EGFR*-wt human lung adenocarcinoma according to cBioPortal Lung Adenocarcinoma (TCGA, 2014) data set. (B) CCL22 protein levels in the culture supernatant of two *EGFR*-mt LA cell lines after EGFR-TKI (AZD9291) treatment were compared using BioPlex assay kit.
* p<0.05

(A)

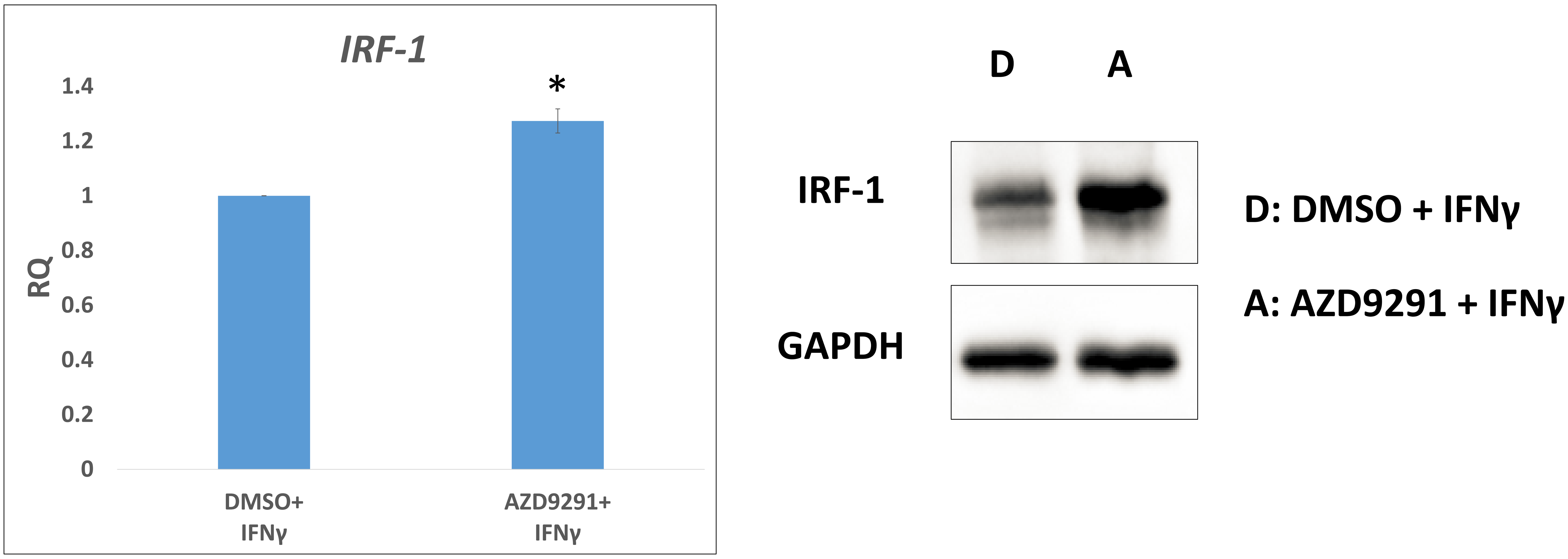

(B)

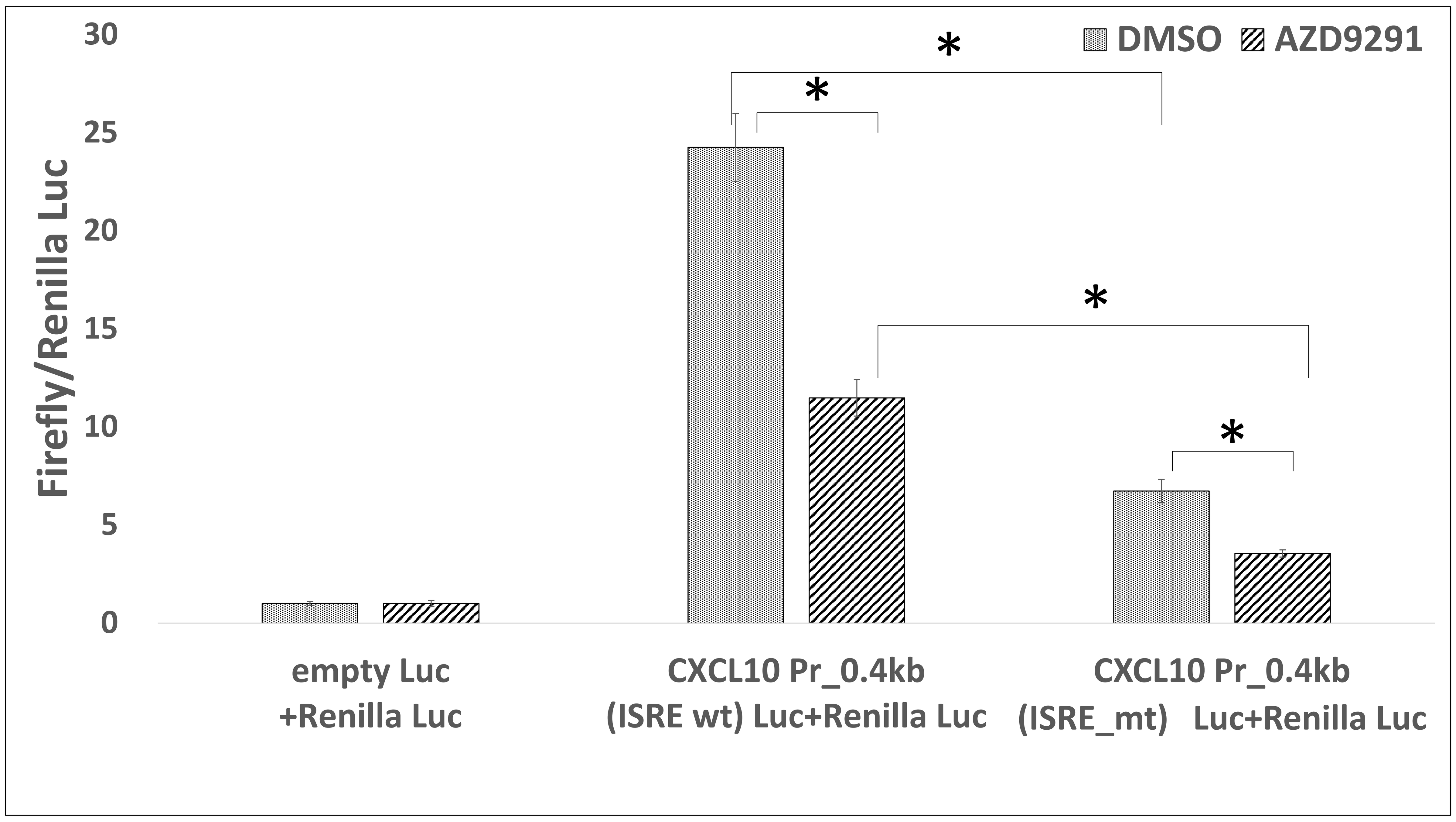

Fig. S7 The effect of EGFR-TKI on the IRF-1 expression level and promoter activity of ISRE. (A) RT-qPCR of *IRF-1* with or without AZD9291, EGFR-TKI, in HCC827 cell line. (Left panel) * $p<0.05$. Immunoblot of IRF-1 of HCC827 cell line with or without AZD9291. GAPDH is a loading control. (Right panel) (B) Luciferase assay with 0.4kb-*CXCL10* promoter-firefly luciferase plasmid vectors with or without site-directed mutagenesis in the ISRE site (wt; aaagtgaaa, mt; aGagtgaGa) in HCC827 cells. Baseline (DMSO) firefly luciferase activity decreased with mt ISRE promoter. AZD9291 decreased the promoter activity in both wt and mt ISRE promoters. Firefly luciferase activity was normalized for transfection efficiency by Renilla luciferase activity. Biological quariplicates. The values were also normalized by those of empty (promoterless) firefly luciferase vector. * $p<0.005$

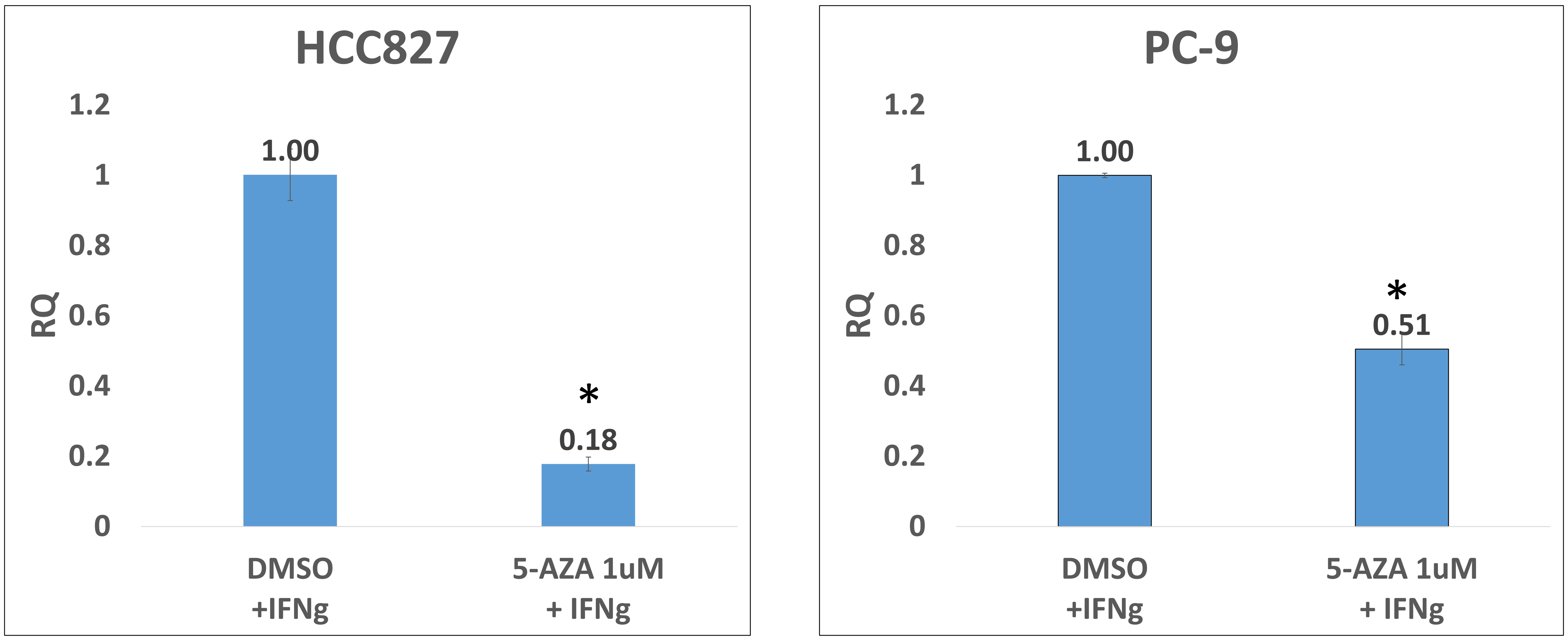

Fig. S8 The effect of 5-azacytidine (5-AZA) on the *CXCL10* mRNA expression level in two *EGFR*mt LA cell lines. qRT-PCR of *CXCL10* with or without 5-AZA, DNA methyltransferase inhibitor, in HCC827 (Left panel) and PC-9 (Right panel). * $p<0.005$

Full length blots of Fig. 4D

High exposure to visualize MWM

<Histone H3ac Ab>

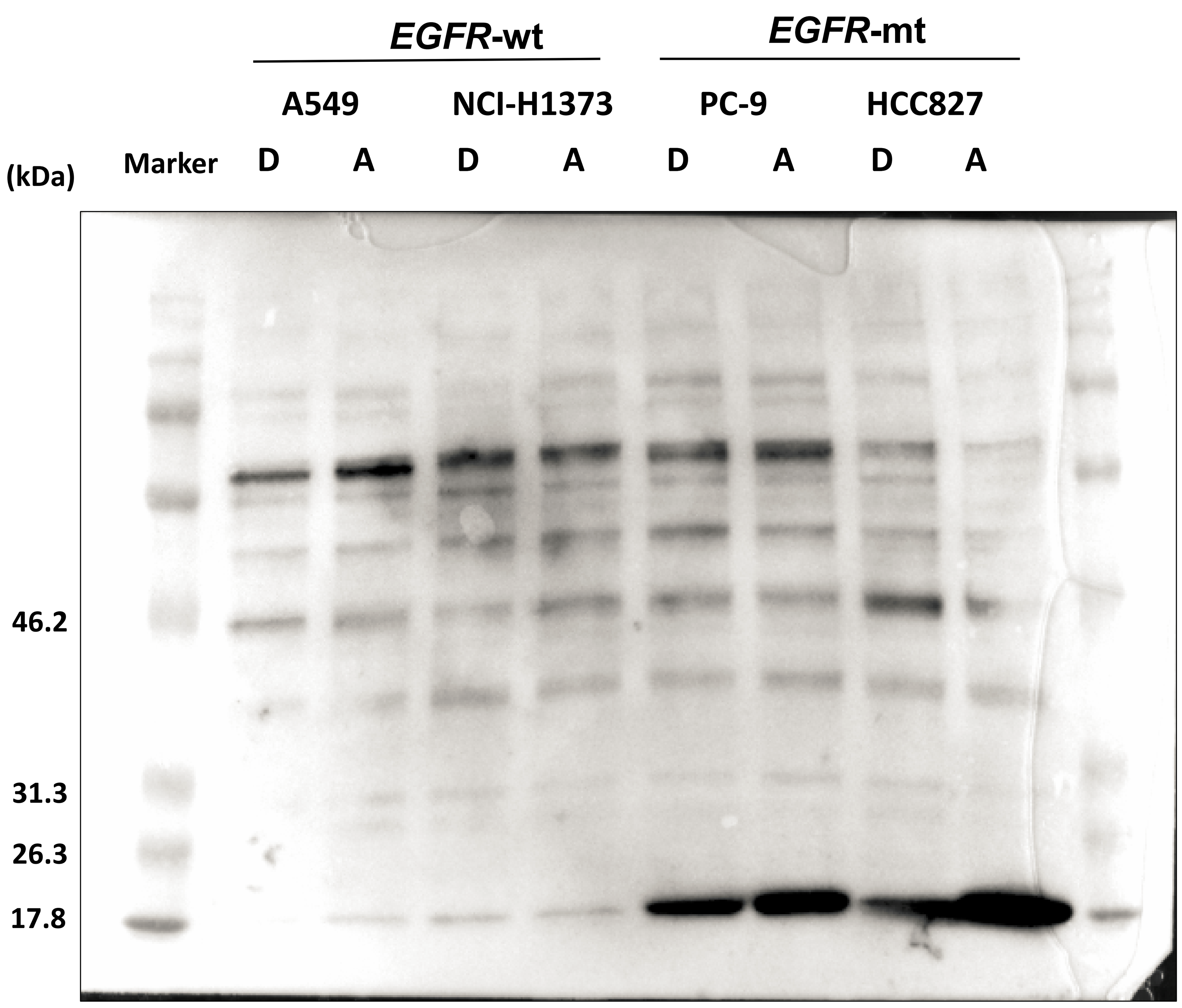

D: DMSO
A: AZD9291

Histone H3; 17kDa

<Lamin A/C Ab>

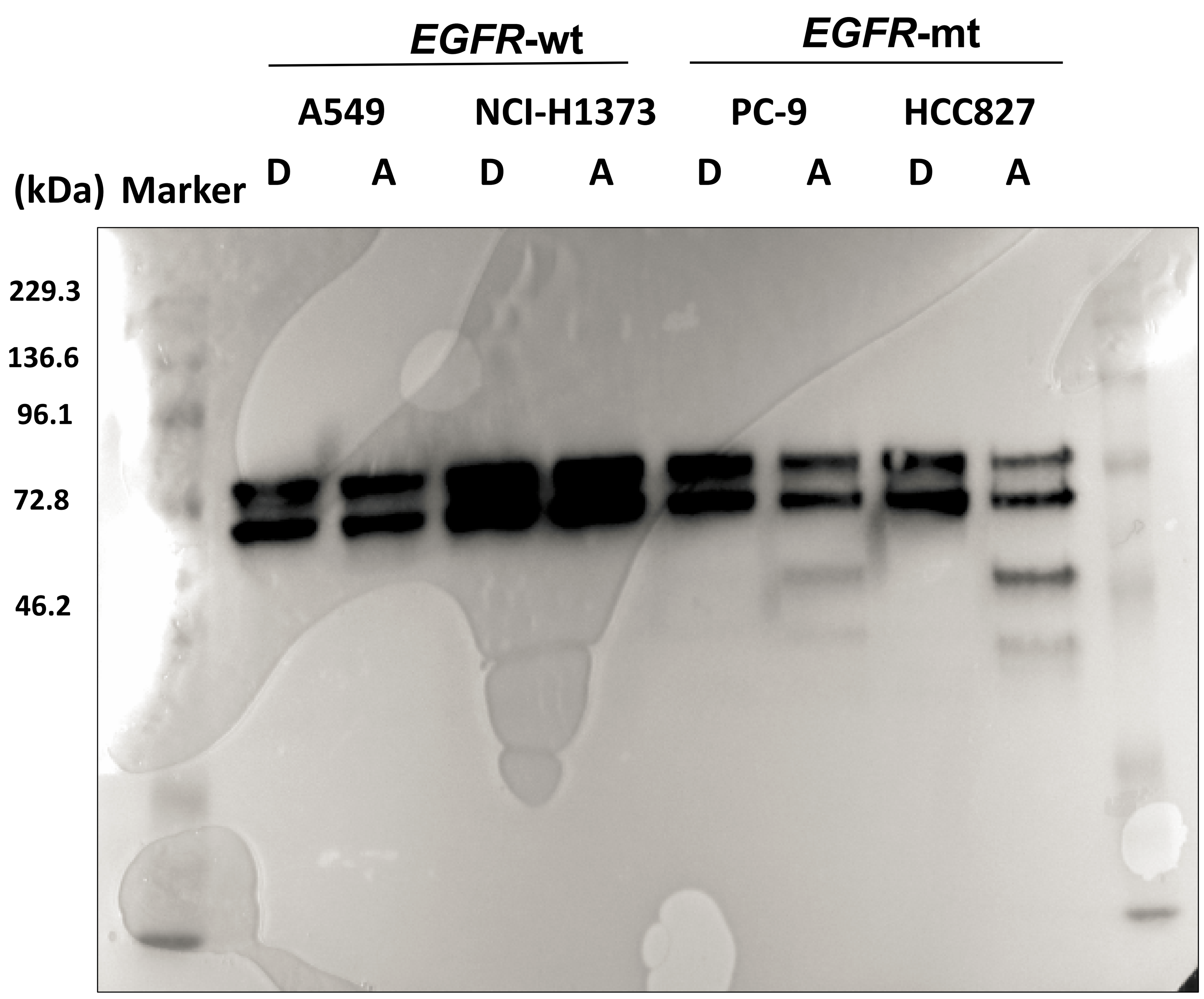

Lamin A/C; 75kDa

Low exposure

<Histone H3ac Ab>

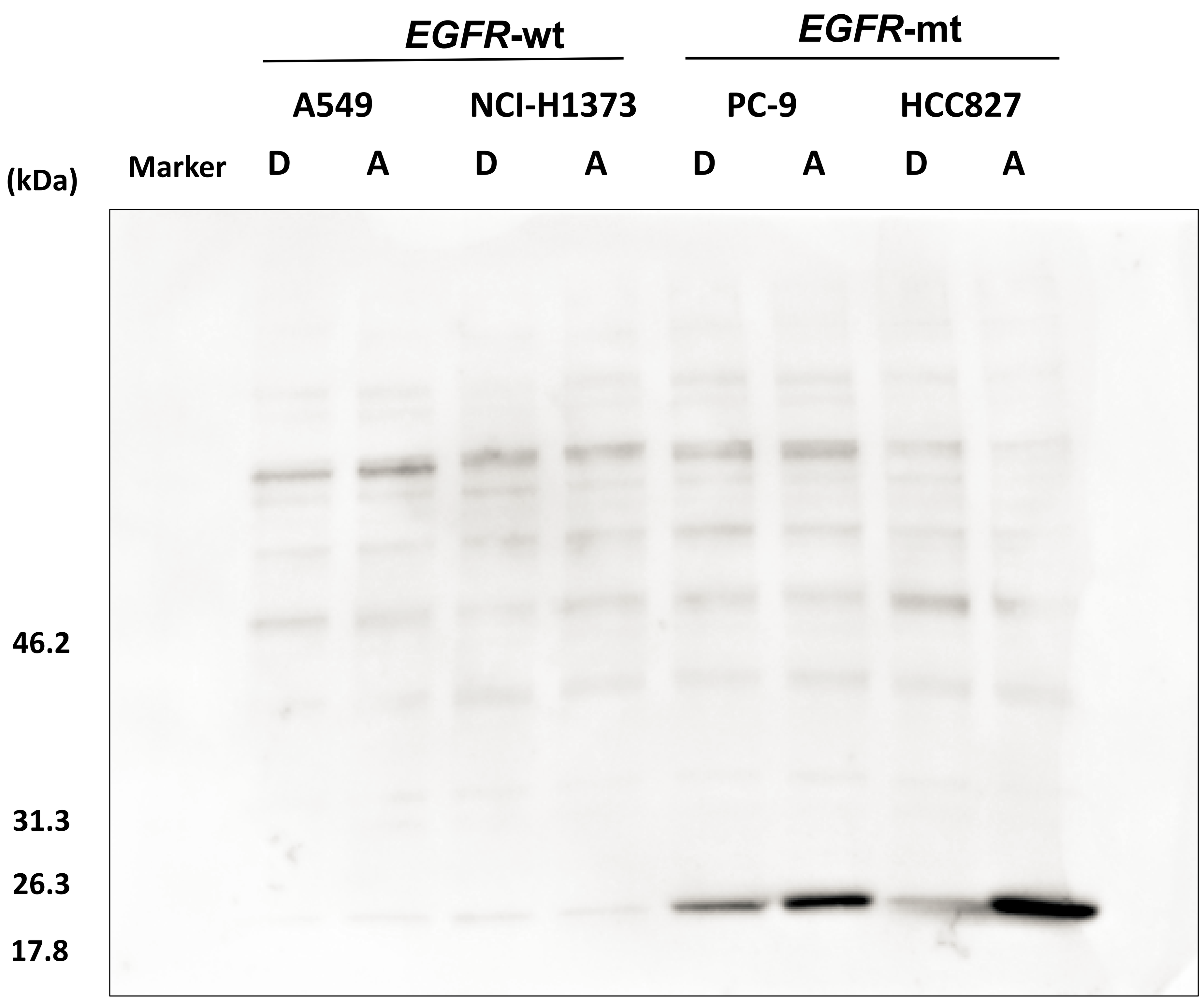

D: DMSO
A: AZD9291

Histone H3; 17kDa

<Lamin A/C Ab>

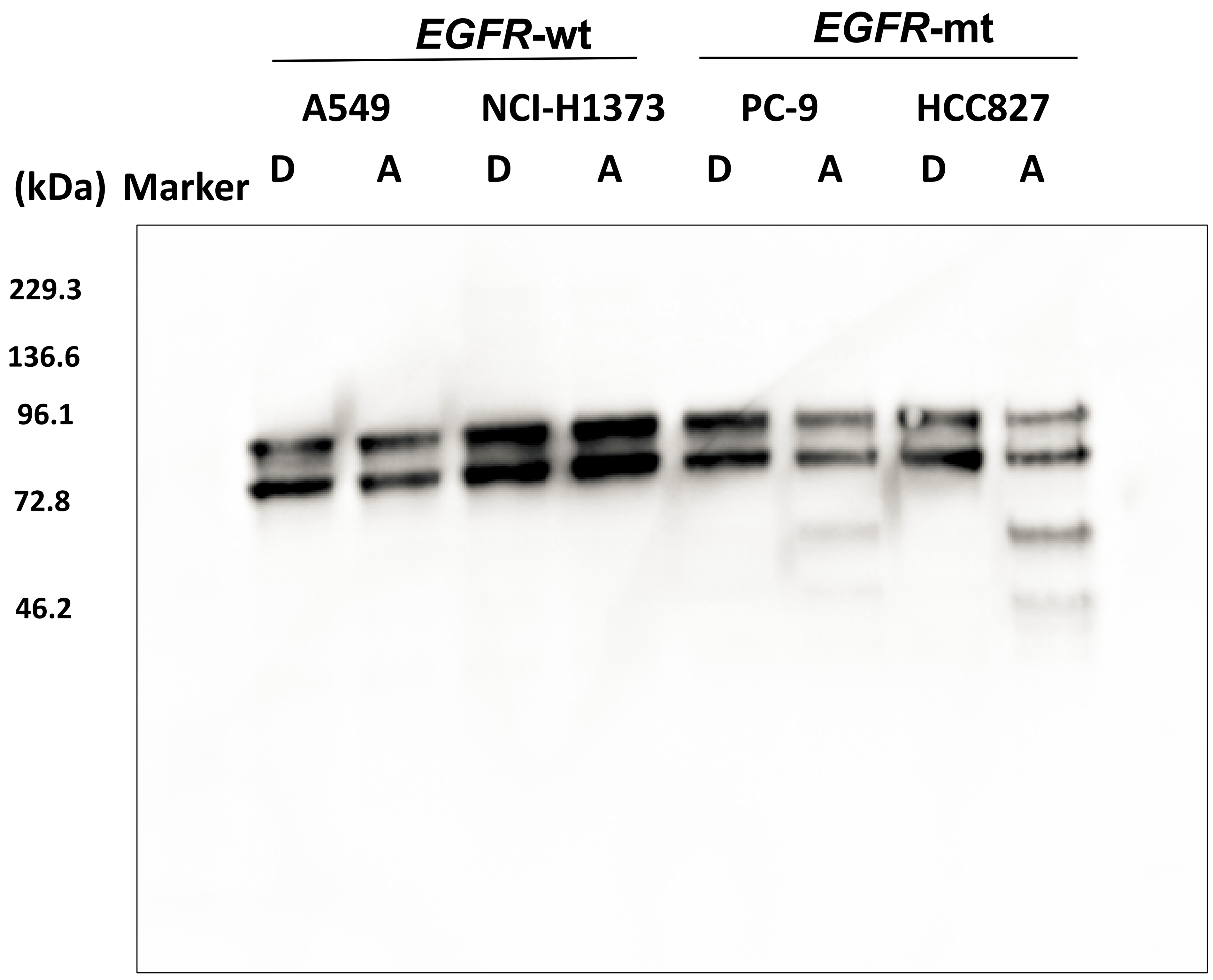

Lamin A/C; 75kDa

Full length blots of Fig. S7A

High exposure to visualize MWM

<IRF-1 Ab>

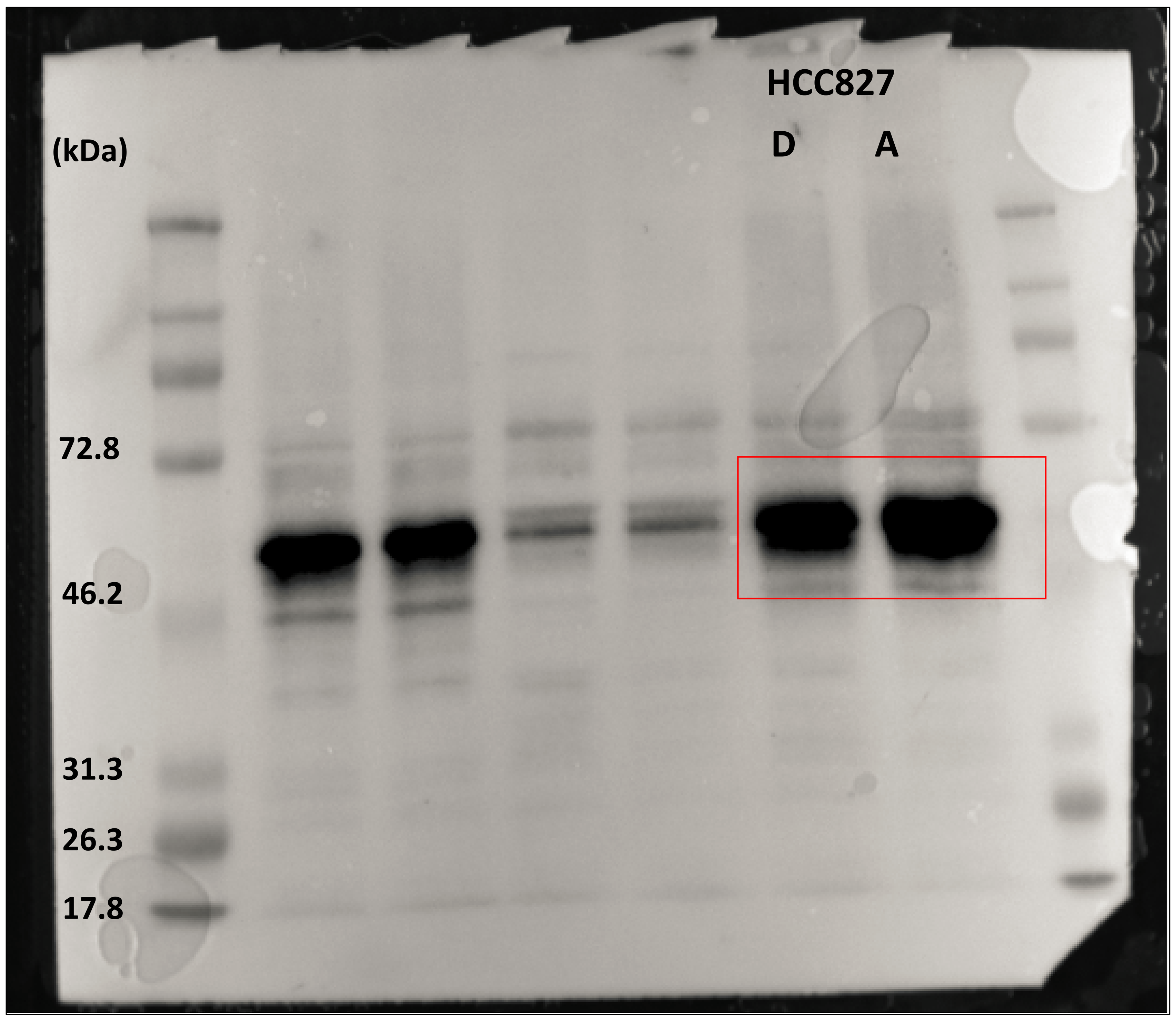

<GPPDH Ab>

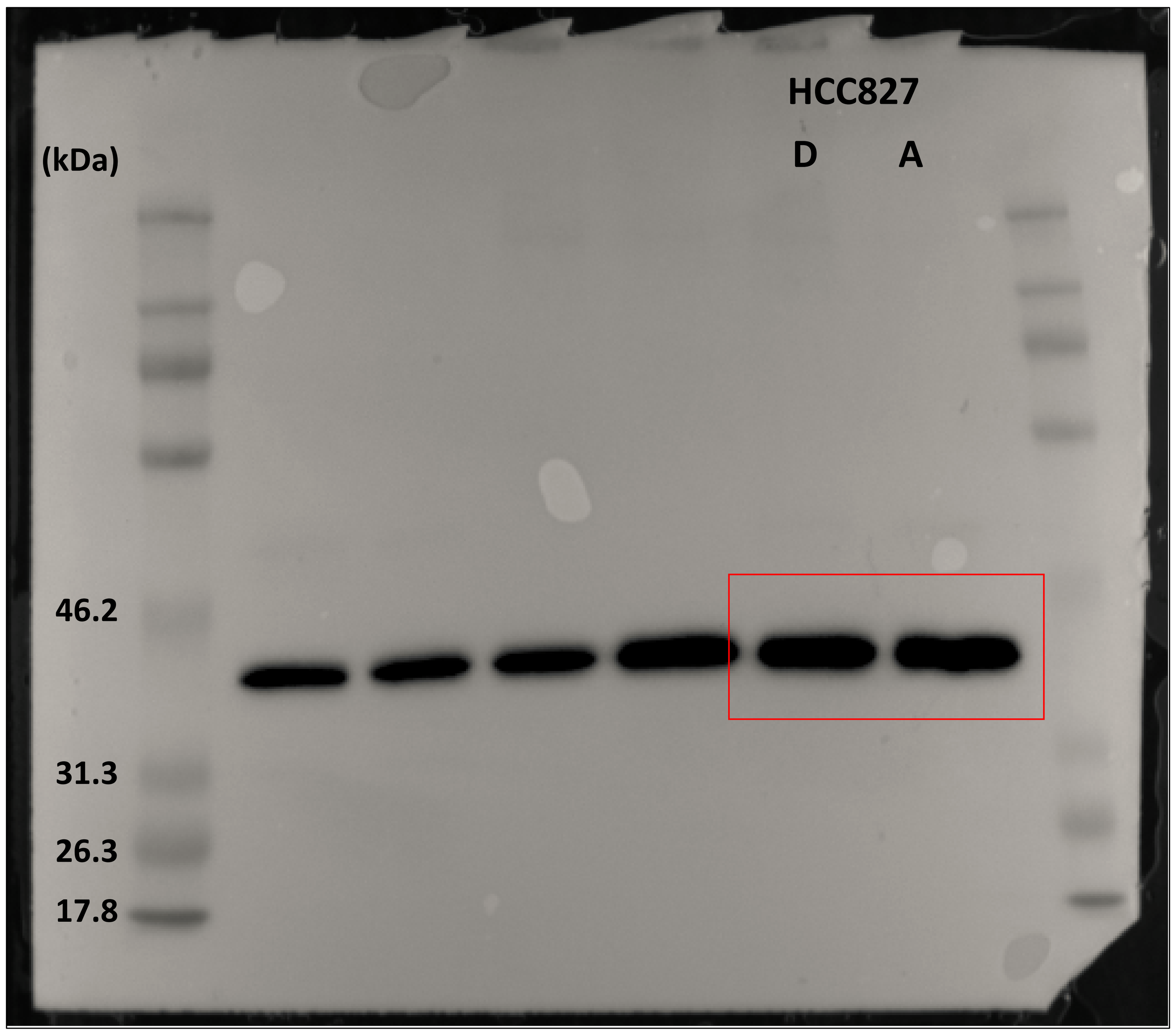

D: DMSO
A: AZD9291

IRF-1; 45-48kDa

GAPDH; 37kDa

Low exposure

<IRF-1 Ab>

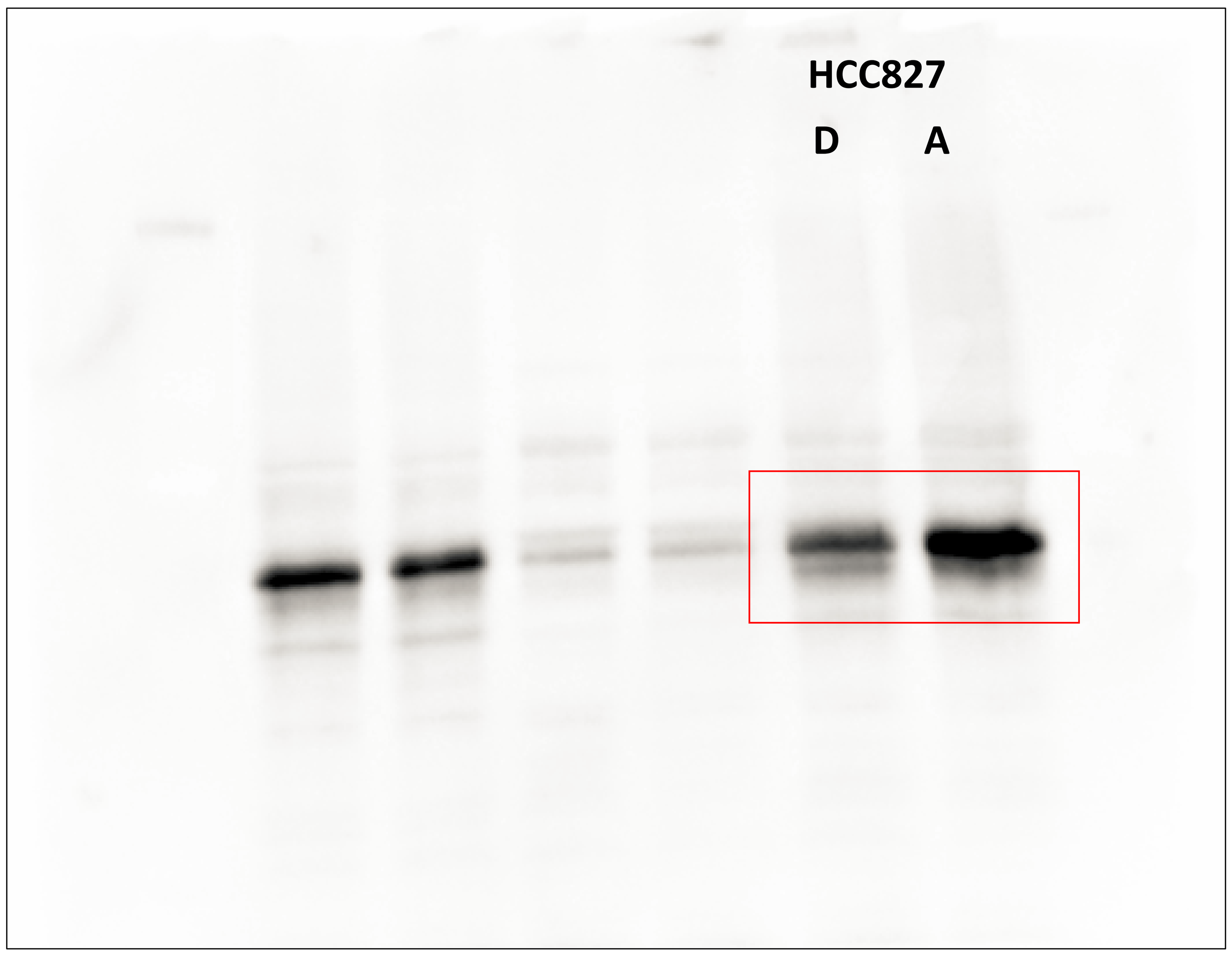

<GPPDH Ab>

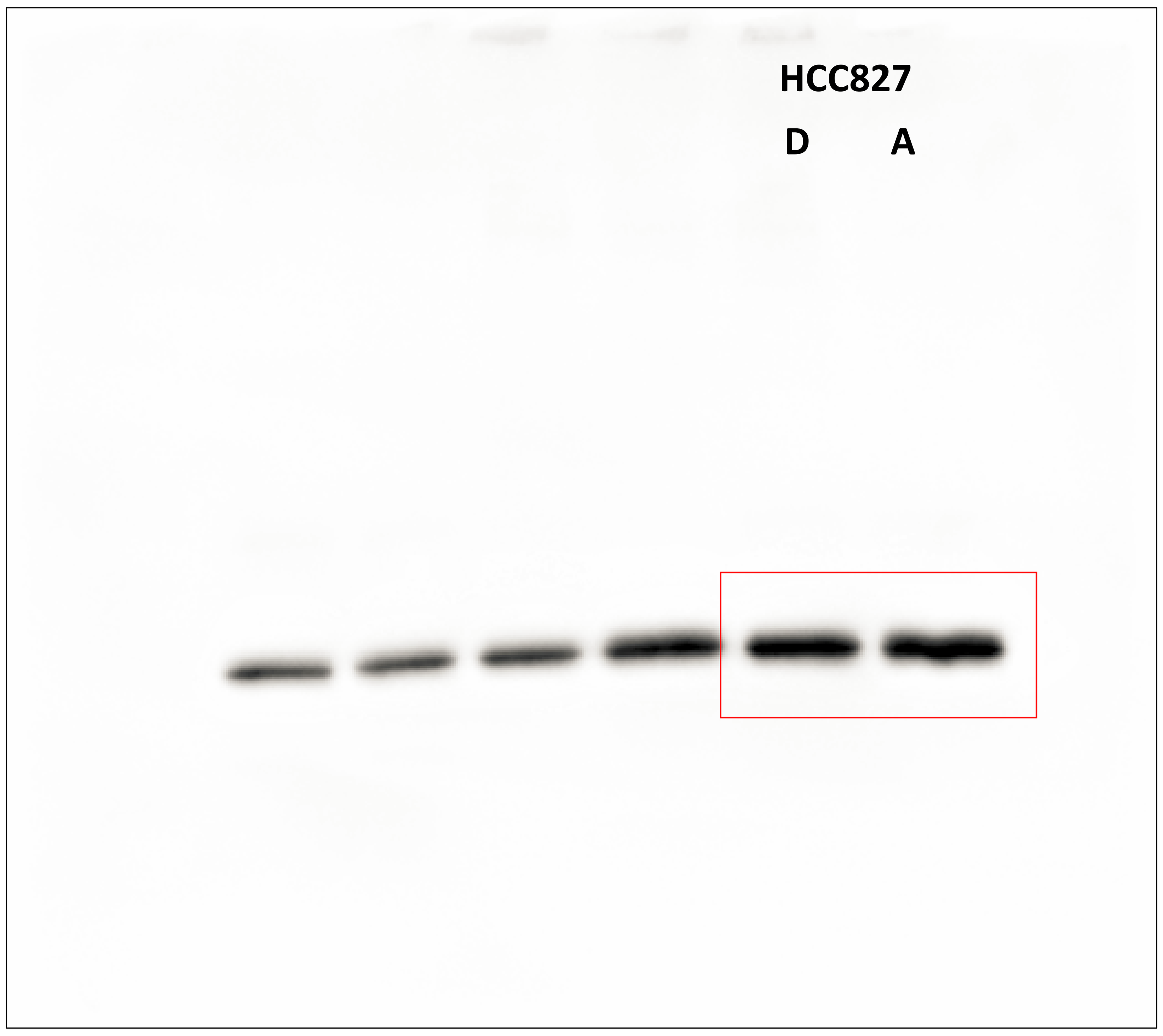

Cropped part in Fig. S7A
